# Synthesis of Click-Ready Aminooxy-Terminated Poly(ε-caprolactone) Oligomers for Oxime Ligation

**DOI:** 10.1021/acs.bioconjchem.6c00091

**Published:** 2026-04-23

**Authors:** Weilin Zhang, Karin Odelius, Peter Olsén

**Affiliations:** † Wallenberg Wood Science Center, Department of Fibre and Polymer Technology, KTH Royal Institute of Technology, Stockholm 10044, Sweden; ‡ Wallenberg Wood Science Center, Laboratory of Organic Electronics, 4566 Linköping University, Norrköping 60174, Sweden

## Abstract

Oxime ligation, a chemoselective coupling between carbonyl compounds and aminooxy groups, enables conjugation under mild and aqueous conditions. Here, we integrate this reaction with degradable polymers through a one-pot synthesis of aminooxy-functionalized poly­(ε-caprolactone) (PCL) oligomers. The bifunctional initiator 6-(*tert*-butyloxycarbonylaminooxy)-1-hexanol was employed for simultaneous ε-caprolactone ring-opening polymerization and in situ Boc-deprotection catalyzed by methanesulfonic acid (MSA). Optimized conditions afforded >95% conversion for both reactions, while maintaining well-defined oligomer structures (DP = 5–20). The resulting NH_3_^+^–O–PCL oligomers underwent rapid and efficient oxime ligation with a diverse set of aldehydes and ketones, achieving >90% conversion within 1–2 h at room temperature. Reactions proceeded effectively even in mixed aqueous solvents (H_2_O/MeOH/CHCl_3_ = 1:3:1), for example, with the reducing ends of glucose and xylose. This work establishes a straightforward, water-tolerant synthetic platform for preparing “click-ready” degradable polymers, enabling broad integration with biobased and functional substrates.

## Graphical abstract

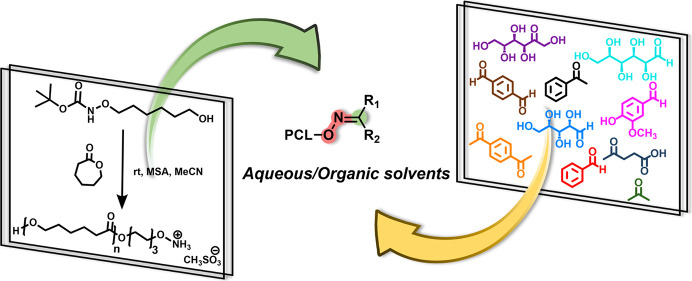

## Introduction

The combination of degradable polyesters with hydrophilic, biobased substrates, such as natural fibers and proteins, is important for advancing sustainable materials and targeted therapeutics. A major obstacle, however, is that these substrates inevitably contain water,^1–4^ while the synthesis of many degradable polyesters relies on ring-opening polymerization (ROP), a method highly sensitive to moisture.^5,6^ Residual water readily initiates polymerization and competes with intended initiating sites,^7,8^ limiting the applicability of direct ROP-based approaches to water-containing substrates.

An alternative strategy is to presynthesize the degradable polymer and couple it to the biobased substrate using chemoselective, water-tolerant reactions.^9–12^ Among degradable polymers, poly­(ε-caprolactone) (PCL) is particularly attractive due to its biocompatibility in certain applications,^13,14^ ease of processing,^15^ and slow but predictable degradation.^16^ PCL is mainly synthesized via ROP of ε-caprolactone (ε-CL), enabling precise control over the molecular weight and end-group functionality. PCL is polymerizable with a wide range of catalysts and is thermodynamically strongly favored toward its polymer form,^17^ making it a robust platform for functionalization and coupling reactions. Various functional groups, including halogens,^18–20^ alkynes,^21,22^ hydroxyl,^23^ sulfonyl moieties,^24^ amino group,^25,26^ and carboxylic acids,^27^ have been introduced into ε-CL structure while maintaining polymerizability. However, functionalized monomers often require multistep synthetic routes, limiting scalability and broader applicability. Another key consideration is the degree of functionality: for selective postpolymer conjugation, particularly those relying on bioorthogonal or click-type reactions, a single, well-defined functional group per polymer chain would be ideal.

When combining degradable polymers with hydrophilic substrates, enforcing strictly anhydrous conditions is hard; instead, a more robust strategy is to design functional degradable polymers that tolerate water during subsequent coupling. Oxime ligation is well suited for this purpose: it proceeds rapidly under mild, aqueous conditions, forms hydrolytically stable linkages,^28^ and is widely used for conjugating polymers to carbonyl-bearing substrates.^29–31^ These features make it an attractive route for integrating degradable polyesters with biobased materials, particularly under green chemistry conditions.^32–34^ Yet its application to synthetic degradable polyesters such as PCL remains largely unexplored.

Inspired by the challenge of creating “click-ready” monofunctionalized degradable polymers, we report here a one-pot synthesis of protonated aminooxy-terminated PCL oligomers (NH_3_^+^–O–PCL) using *tert*-butyloxycarbonyl-6-aminooxy-1-hexanol as an initiator and methanesulfonic acid (MSA) as a dual catalyst for both ROP of ε-CL and *tert*-butyloxycarbonyl (Boc) group deprotection. This strategy yields end-functionalized PCL oligomers that are readily compatible with oxime ligation. As a proof of concept, we evaluated the ligation efficiency of NH_3_^+^–O–PCL with a series of model carbonyl-containing compounds to assess the reactivity, solvent compatibility, and coupling efficiency under mild and aqueous-compatible conditions. This study focuses on the fundamental reactivity between NH_3_^+^–O–PCL and diverse aldehyde and ketone substrates, aiming to provide a clear framework for the future use of oxime ligation in more complex, water-compatible hybrid materials.

## Experimental Section

### Materials

All materials and chemicals were used as received without further purification: N-Boc-hydroxylamine (≥98%, Sigma-Aldrich), 6-bromo-1-hexanol (97%, Sigma-Aldrich), ε-caprolactone (synthesis grade, Sigma-Aldrich), D-(+)-glucose (ACS reagent, Sigma-Aldrich), D-(+)-xylose (ACS reagent, Sigma-Aldrich), D-(−)-fructose (≥99%, Sigma-Aldrich), methanesulfonic acid (MSA, ≥99%, Sigma-Aldrich), 1,8-diazabicyclo (5.4.0) undec-7-ene (DBU, 98%, Sigma-Aldrich), 1-hexanol (98%, Sigma-Aldrich), acetone (≥99%, VWR), benzaldehyde (≥99%, Sigma-Aldrich), terephthalaldehyde (98%, Sigma-Aldrich), vanillin (99%, Sigma-Aldrich), acetophenone (99%, Sigma-Aldrich), levulinic acid (99%, Sigma-Aldrich), 1,4-diacetylbenzene (99%, Sigma-Aldrich), magnesium chloride (MgCl_2_, Sigma-Aldrich), acetonitrile (MeCN, ≥95%, VWR), dichloromethane (CH_2_Cl_2_, 99%, VWR), chloroform (CHCl_3_, ≥99.8%, VWR), sodium chloride (NaCl, ≥98%, VWR), triethylamine (≥99.5%, Carol Erba), and 3,5-diaminobenzoic acid (DABA, 98%, Lancaster), chloroform-d (CDCl_3_, Sigma-Aldrich), and dimethyl sulfoxide-d_6_ (DMSO-*d*_6_, VWR). No unexpected or unusually high safety hazards were encountered.

### Instruments

#### Nuclear Magnetic Resonance (NMR)

Nuclear magnetic resonance (NMR) was used to determine chemical composition, monomer conversion, and the conversion of ligation. ^1^H NMR (400 HMz), ^13^C NMR (100 MHz), correlation spectroscopy (COSY), and heteronuclear single quantum coherence (HSQC) were recorded at room temperature using a Bruker advance III HD (400 MHz) spectrometer with CDCl_3_ or DMSO-*d*_6_ as the solvent.

#### Size Exclusion Chromatography (SEC)

Size exclusion chromatography (SEC) was used to monitor the molecular weight and dispersity (Đ) of PCL oligomers and ligation products. A Malvern GPCMAX equipped with an autosampler and three PLgel 5 μm columns (guard column 7.5 × 50 mm and mixed-D columns 300 × 7.5 mm) was utilized. The calibration curve was created using polystyrene standards with narrow dispersity in the range 162–364,000 g/mol. The samples were analyzed with CHCl_3_ as eluent containing 2% (v/v) toluene as an internal standard, at a flow rate of 0.5 mL/min using a refractive index (RI) detector.

#### Attenuated Total Reflectance Fourier Transform Infrared (ATR-FTIR)

Attenuated total reflectance Fourier transform infrared (ATR-FTIR, PerkinElmer Spectrum 100) was used to analyze the functional groups of the synthesized compounds and to obtain structural information. The FTIR was equipped with a Golden Gate diamond ATR (Gaseby Specac Ltd., UK), and spectra were recorded in the range of 4000–600 cm^–1^ using 8 consecutive scans at room temperature with a resolution of 8 cm^–1^.

#### Electrospray Ionization Mass Spectrometry (ESI-MS)

Measurements were carried out using a Bruker amaZon speed ESI-ion trap-ETD Mass Spectrometer (Bruker Daltonik GmbH, Bremen) with a nebulizer gas (N_2_) pressure of 0.5 bar. The capillary and end plate offset voltages are 2200 and 500 V, respectively (positive mode). For the drying gas, N_2_ was used at a flow rate of 5 L/min and a temperature of 150 °C. Data were acquired over the range *m*/*z* 50–1500 with an accumulation time of 30–40s and evaluated by Compass Data Analysis.

### Procedure

#### Synthesis of 6-(*Tert*-Butyloxycarbonylaminooxyl)-1-hexanol

DBU (2.69 mL, 18 mmol) was added dropwise over 5 min to a solution of N-Boc-hydroxylamine (2.00 g, 15 mmol) and 6-bromohexan-1-ol (1.96 mL, 15 mmol) in CH_2_Cl_2_ (80 mL). The reaction mixture was allowed to stir for 24 h at room temperature. The resulting solution was transferred to a separating funnel with CH_2_Cl_2_ (50 mL). The solution was washed with 1 M HCl (100 mL) and brine (100 mL). Then, the combined organic layer was dried with MgCl_2_ and concentrated.^35,36^ The resulting pale yellow oil was purified by column chromatography (EtOAc:heptane, 1:1) with an isolated yield of 40%s.

#### Deprotection of 6-(*Tert*-Butyloxycarbonylaminooxyl)-1-hexanol

The synthesized 6-(*tert*-butyloxycarbonylaminooxyl)-1-hexanol (100 mg, 0.043 mmol) was dissolved in CDCl_3_. Two parallel samples were prepared with 1.1 and 2.0 equiv of MSA, which was added separately. Then, the Boc deprotection process was monitored by ^1^H NMR spectroscopy over time.

#### One-Pot Synthesis of Oligomers DP5, DP10, DP20, and DP50

The Boc-protected initiator 6-(*tert*-butyloxycarbonylaminooxyl)-1-hexanol (Boc-I, 100 mg, 0.043 mmol) and ε-CL (5, 10, or 20 equiv) were added to 20 mL vials for the synthesis of 6-(aminooxy)­hexan-1-ol methanesulfonate-terminated PCL (PCL–(CH_2_)_6_–ONH_3_^+^·CH_3_SO_3_^–^) and denoted DP5, DP10, DP20, and DP50 oligomers. The ROP and deprotection of the Boc group were performed simultaneously with MSA (from 1.0 to 3.0 equiv, with increments of 0.5 equiv) as the catalyst for polymerization and the reagent for deprotection in MeCN (1 mmol/mL) at room temperature, followed by precipitation of the synthesized oligomers in diethyl ether. The molecular weights of the oligomers were monitored by ^1^H NMR and SEC analysis. The conversion of deprotection and ROP was calculated from the ^1^H NMR spectra based on the integral signals from the methylene group of ε-CL (−O–CH_2_–, δ = 4.21 ppm), the methylene group of PCL repeating units (−CH_2_–CO–, δ = 4.06 ppm), the end-group of PCL oligomer (−CH_2_OH, δ = 3.65 ppm) and the methyl groups of Boc group ((CH_3_)_3_–C–, δ = 1.48 ppm).

$$\mathrm{Conversion}\;(\%)=\frac{I_{4.06}}{I_{4.06}+I_{4.21}}\cdot 100\%$$

$$\mathrm{Deprotection}\;(\%)=\frac{I_{1.48}}{I_{1.48,\mathrm{initial}}}\cdot 100\%$$

For the oligomers, the methylene group linked to the protonated aminooxy groups (*e′* of initiator, 4.06–4.09 ppm, Figure 1b) overlapped with the methylene group attached to the oxygen atom in the PCL repeating unit (4.06 ppm, Figure S4). After ligation with aldehydes/ketones, these (*e*′**) signals separated from PCLs repeating unit signal (δ = 4.06 ppm) with a chemical shift at 4.00 or 4.18 ppm (*e′*), depending on which substrate the oligomer was ligated to. Acetone was selected as the ligation model due to its simple structure and high ligation conversion. The fraction of ROP initiated by the −OH end of initiator (I–OH) estimated by spectra of DP5-A, DP10-A, and DP20-A (Figure S6), and the fraction of I–OH-initiated ROP chains (ROP_I_) was expressed as

$${\mathrm{ROP}}_{I}=\frac{I_{4.00}}{I_{3.65}}\cdot 100$$

**Figure 1:**
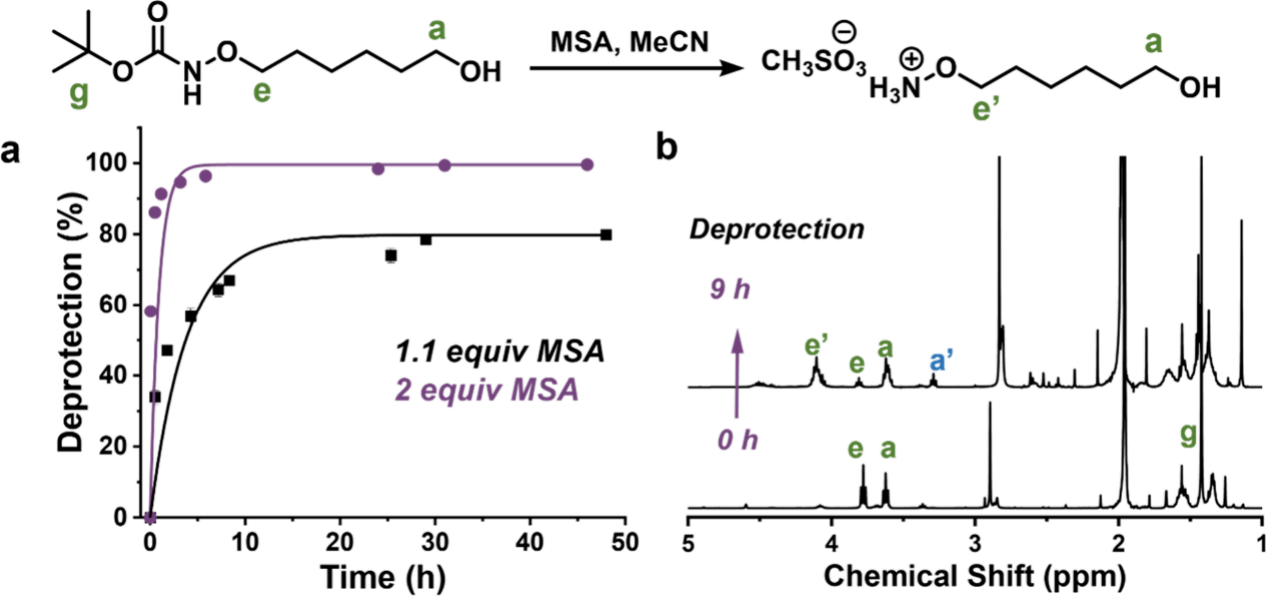
(a) Conversion of the deprotection of the Boc group at 1.1 and 2.0 equiv of MSA over time. (b) ^1^H NMR spectra (400 MHz, CDCl_3_) of the Boc-I at 0 and 9 h of deprotection.

### Sequential Synthesis of Oligomer DP5_sequential_

In the first step, 100 mg (0.043 mmol) of 6-(*tert*-butyloxycarbonylaminooxyl)-1-hexanol and 3 equiv of MSA were dissolved in the CHCl_3_ to remove the Boc group overnight at room temperature. Then, 5 equiv of ε-CL was added to the solution to perform the ROP for 8 h as the second step, followed by precipitation of the synthesized oligomers in diethyl ether.

#### Ligation in Organic Solvent

Acetone, vanillin, benzaldehyde, terephthaldehyde, 1,4-diacetylbenzene, and acetopheone were selected to investigate the ligation ability in organic solvent. DP5, DP10, and DP20 (1 equiv, 0.04 mmol) were placed in vials, respectively, and dissolved in CHCl_3_ (1 mL) under stirring. Then, the selected aldehyde- or ketone-containing compounds (1 equiv of carbonyl group to the oligomer) and TEA (4–7 μL, the oligomers were predissolves in the mixed aqueous solvents to observe the pH.) were added to the solution to tweak the solution to be weakly acidic. The solution was stirred for 8 h at room temperature for the oxime linkage formation. For the ligation efficiency calculation between PCL oligomers and various carbonyl compounds, the peak of the PCL end-group at 3.65 ppm was selected as the standard, and the integral value was normalized. The target proton peaks (the proton connected to the carbonyl carbon or the methyl protons adjacent to the carbonyl group, shown in Table S1 and Figures S7–S12) were selected as signals for analysis of conversion. The *I* is the integral value of the target group peak after ligation, and *I*_0_ is the integral value of the target group at 100% conversion.

$$\mathrm{Conversion}\;\mathrm{of}\;\mathrm{ligation}\;(\%)=\frac{I}{I_{0}}\cdot 100\%$$

For the selected aldehydes and ketones, the ^1^H NMR chemical shifts of the target proton peaks are as follows: DP5-A: 1.86 ppm; DP5-V: 7.98 ppm; DP5-B: 8.07 ppm; DP5-T: 8.07, 8.13 ppm; DP5-D: 2.22, 2.24 ppm; and DP5-P: 2.22 ppm.

#### Ligation in Mixed Aqueous Solvents

For the ligation, DP5, DP10, and DP20 (1 equiv, 0.04 mmol) were dissolved in vials with a mixture of solvents (CHCl_3_/MeOH/H_2_O, volume ratio 1:3:1), respectively. 3,5-Diaminobenzoic acid was added as a catalyst (6.39 mg, 1 equiv, 0.04 mmol) and TEA (4–9 μL) to tweak the pH to around 4–5 for the ligation. The reducing sugar (1 equiv of carbonyl groups for the oligomers) was added to the solution to react at room temperature. Upon completion, the reaction mixture was extracted with dichloromethane (CH_2_Cl_2_) and washed sequentially with saturated aq. NaHCO_3_, brine, and dried (MgCl_2_). The same method was used for all monosaccharide carbohydrates. ^1^H NMR spectra are shown in Figures S13–S18. The methylene group of DP5 (δ = 2.26 ppm) was normalized to the reference integral. As for monosaccharides molecules, all NMR experiments were run in DMSO-*d*_6_ due to the poor solubility in CDCl3. The DP value of DP5 (δ = 4.00 ppm) obtained from CDCl_3_ (2.3.2) was used as a calibration standard for integrating the spectra in DMSO-*d*_6_. The sum of integral signals at 7.32 ppm (E), 6.69 ppm (Z),^37^ and 5.19 ppm (β)^38^ represents the tautomerism of the ligation products with glucose/xylose. Based on this method, conversion of ligation of the carbohydrates was evaluated by comparing the integrals *I*_sum_ and *I*_0_, and *I*_0_ is the theoretical integration for 100% conversion.

$$I_{\mathrm{sum}}=I_{7.32\mathrm{ppm}}+I_{6.69\mathrm{ppm}}+I_{5.19\mathrm{ppm}}$$

$$\mathrm{Conversion}\;\mathrm{of}\;\mathrm{ligation}\;(\%)=\frac{I_{\mathrm{sum}}}{I_{0}}\cdot 100\%$$

For the selected aldehydes and ketones, the ^1^H NMR chemical shifts of the target proton peaks are as follows: DP5-A_2_: 1.86 ppm; DP5-V_2_: 7.98 ppm; DP5-L: 1.86 ppm; DP5-X: 7.35, 6.70, 5.16 ppm; DP5-G: 7.30, 6.67, 5.18 ppm; and DP5-F: /.

## Results and Discussion

### One-Pot Synthesis of Aminooxy-Terminated PCL Oligomers

Oxime ligation, a chemoselective coupling between carbonyl compounds and aminooxy groups, offers a versatile strategy for constructing functional materials under mild conditions, even in the presence of water. Here, we explore this ligation method as a route to conjugate poly­(ε-caprolactone) (PCL) with a variety of aldehydes and ketones. To enable this, the bifunctional compound 6-(*tert*-butyloxycarbonylaminooxyl)-1-hexanol (Boc-I) was synthesized to serve as an initiator for the ROP of ε-CL from its hydroxyl group, while the aminooxy group offers a reactive site for subsequent oxime ligation. Commercially available N-Boc-hydroxylamine (2.00 g, 15 mmol) was used together with 6-bromohexan-1-ol (1.96 mL, 15 mmol) in DCM to prepare the Boc-I, following previously published procedures.^35,36^ Since Boc deprotection proceeds under acidic conditions, we envisioned the preparation of protonated aminooxy-terminated PCL oligomers via a one-pot strategy. Strong Bro̷nsted acids, such as hydrochloric acid (HCl, p*K*_a_ ≈ −7.0),^39^ trifluoroacetic acid (TFA, p*K*_a_ ≈ −0.2),^40^ and methanesulfonic acid (MSA, p*K*_a_ ≈ −1.9),^39^ should be sufficiently acidic to facilitate Boc deprotection, while simultaneously catalyzing ROP. However, combining both steps in a one-pot strategy is fundamentally challenging, as the acid must be strong enough to cleave the Boc group and directly protonate the aminooxy initiator (RONH_2_ → RONH_3_^+^), to eliminate its nucleophilicity thereby hindering ROP initiation. Among these, MSA offers a promising compromise, being less acidic, and showing good catalytic activity for controlled ROP.^41,42^ To evaluate the activity of MSA, we monitored Boc deprotection by ^1^H NMR using MeCN as an internal standard. The methylene group resonances shifted (e: 3.78 → 4.06 ppm), while the *tert*-butyl resonance (g, δ = 1.48 ppm) gradually decreased, indicating cleavage of the Boc group (Figure 1b).

With 1.1 equiv of MSA, the reaction reached ∼ 80% conversion after 48 h, whereas increasing the amount of MSA to 2.0 equiv resulted in a rapid and near-quantitative conversion (>95%) within 8 h (Figure 1a). Initially, ROP was carried out directly after deprotection (3 equiv of MSA) to study the effect of the sequential addition of monomer targeting a DP5. However, both ^1^H NMR spectra (Figure S27) and SEC (Figure S34) indicate that the initial – OH concentration was reduced during the deprotection step, resulting in much higher *M*_n_ and broader dispersity of DP5_sequential_ than expected. It was found that this was related to ether formation under the strong acidic conditions (peak a′, 3.29 ppm, Figure 1b).

Subsequently, to circumvent the side reaction observed during sequential addition, we conducted a systematic investigation of the one-pot synthesis approach by varying the molar ratio of MSA to initiator from 1.0 to 3.0 equiv to initiator, focusing on its dual role in enabling both Boc-deprotection and catalyzing ROP in MeCN at room temperature, as shown in Figure S4. At 1.0 and 1.5 equiv of MSA, the activity was insufficient to drive both reactions simultaneously, resulting in incomplete conversion even after 7 h. At an MSA loading of 1.5 equiv, the deprotection step was enhanced compared to 1.0 equiv, consistent with the second-order kinetic dependence of Boc-deprotection with MSA previously reported.^43^ Hence, to further optimize the system, we increased the MSA amount from 1.5 to 3.0 equiv in 0.5 equiv increments. For the ROP step, both 2.5 and 3.0 equiv of MSA yielded high monomer conversion (97%) within 1 h. Additionally, the rate of Boc-deprotection continued to increase with higher MSA loading, with 3.0 equiv giving a 66% conversion at 0.5 h compared to 54% for 2.5 equiv. These results confirm that both reactions proceed efficiently under MSA-mediated conditions, supporting the feasibility of the one-pot strategy. Importantly, MSA functions as a catalyst for ROP, but as a reagent for Boc deprotection, emphasizing the need to balance its equivalents for optimal dual functionality. Therefore, 3.0 equiv MSA to initiator was selected as the optimal condition for the one-pot strategy (Figure S4).

With the optimized conditions in hand, oligomers with targeted degrees of polymerization (DP = 5, 10, and 20) were synthesized by varying the molar ratio of ε-CL to initiator during the ROP (Figure 2a). Here, we target low DPs to facilitate the assessment of ligation efficiency in the subsequent step. The chemical structures of the NH_3_^+^–O–oligomers were confirmed by ^1^H NMR spectroscopy (Figures 2b and S4). The kinetics of simultaneous ROP and Boc deprotection were monitored by ^1^H NMR over time. As shown in Figure 2c, under catalysis by 3.0 equiv of MSA, DP5 exhibited the fastest conversion for both ROP and deprotection, reaching >95% conversion within 6 h. All oligomers, regardless of DP, achieved >95% ROP conversion within the same time frame. However, the deprotection rate decreased with increasing DP. This is attributed to a lower MSA-to-monomer ratio at higher DPs, which slows down deprotection. Since MSA-catalyzed ROP of ε-CL proceeds via a monomer-activated mechanism,^42,44^ higher monomer concentrations led to competition between monomer activation and Boc-deprotection, retarding the overall reaction kinetics.

**Figure 2:**
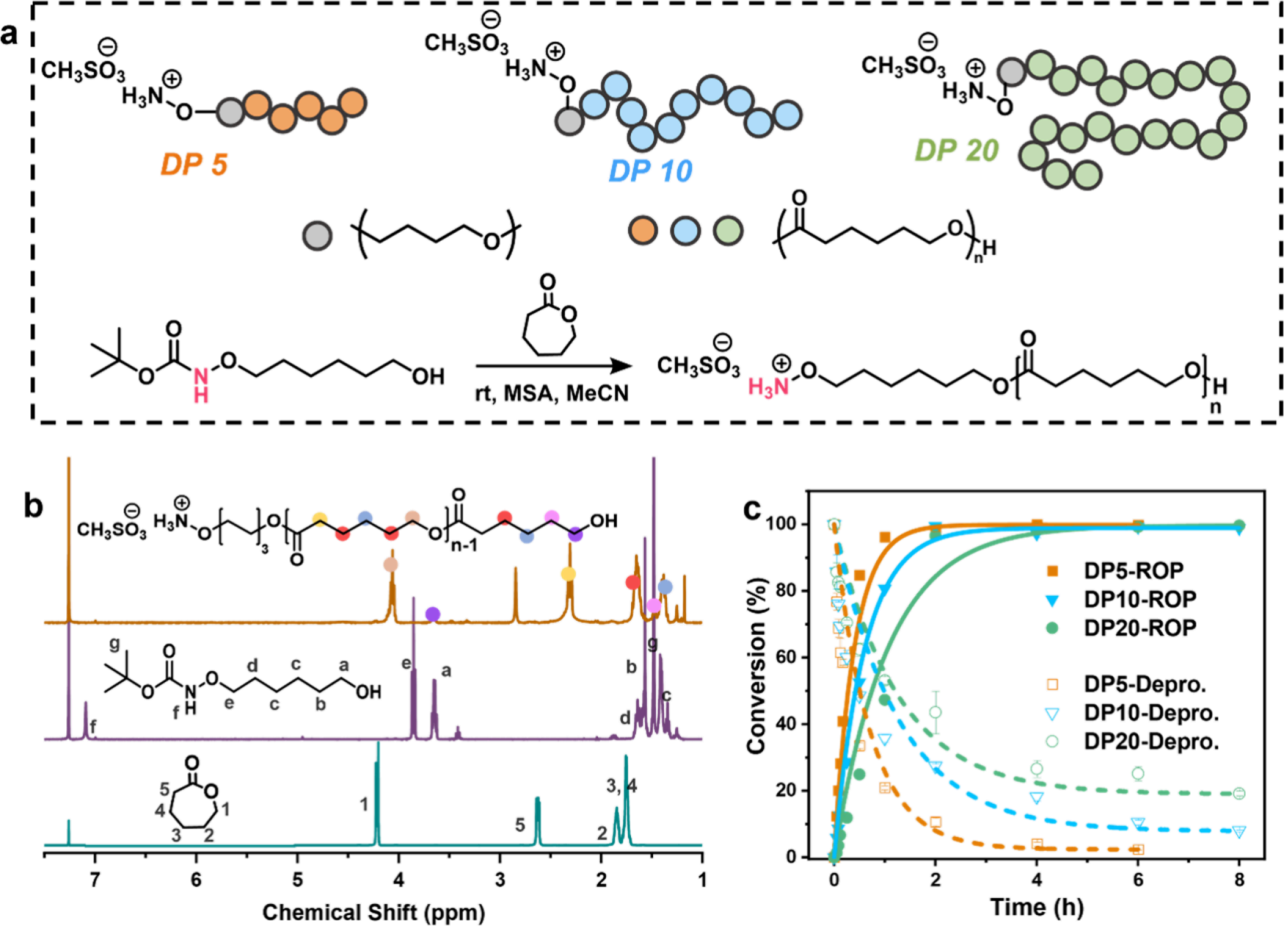
(a) Schematic illustration of the ring-open polymerization and structure; (b) ^1^H NMR spectrum (400 MHz) of the *tert*-butyloxycarbonyl-6-aminooxyl-1-hexanol, ε-CL and DP5 in CDCl_3_; and (c) Conversion of the deprotection and ring-open polymerization at different DP.

To ensure complete deprotection, all polymerizations were extended to overnight reactions. The corresponding ^1^H NMR spectra (Figure S2) show that the *tert*-butyl proton signal from the Boc-group gradually decreased, reaching more than >95% deprotection (Figure S2 and S6). The molecular weight of the synthesized PCL oligomers was determined by both SEC and ^1^H NMR spectroscopy. ^1^H NMR provided consistent values close to the theoretical target, while SEC analysis showed dispersities in the range of 1.5–2.0 (Table S1, Figure S32). Several factors contribute to the slightly broad distribution. First, the extended Boc-deprotection process will broaden the molecular weight distribution during ROP. Second, the charged aminooxy end-group (−O–NH_3_^+^) will affect the hydrodynamic radius (*R*_h_), leading to inaccurate SEC measurements. Third, the inherently low molecular weight of the oligomers approaches the lower detection limits of SEC, where polystyrene calibration standards tend to introduce additional uncertainty.^45^ To obtain more reliable structural information, electrospray ionization mass spectrometry (ESI-MS) and NMR were employed (Figure 3e, Figures S28–S30, and Table S1). The ESI-MS spectra revealed the expected PCL structure, evidenced by a regular mass interval of 114 Da corresponding to the caprolactone repeating unit. Signals at *m*/*z* 116 ([M–NH_3_^+^·]^+^, M = 133 Da) and 156 ([M+CH_3_CN]^+^, M = 115 Da) originate from in-source fragmentation and adduct formation.^46^ These observations are consistent with those of the DP5 structure. Whereas NMR spectroscopy provides more structural insights via end-group analysis (Figures S5 and S22). NMR is particularly well-suited for oligomers with molecular weights below ∼25,000 g/mol^45^. The resulting Mn values are summarized in Table 1. The measured Mn values show small, but consistent, deviations from the theoretical targets. To evaluate the initiation efficiency of the one-pot polymerization process, acetone (A) was used as a model carbonyl for oxime ligation with the protonated aminooxy-terminated oligomers (Table 1 and DP5, DP10, and DP20; Figure S6). ^1^H NMR was used to quantify the degree of ligation, as well as the degree of initiation in the previous step. Both DP5 and DP10 showed high ROP initiation efficiency (>95%), whereas DP20 exhibited lower efficiency (83%). In the one-pot synthesis, the rate of ROP was found to be faster than that of the deprotection rate (Figure 2c). All obtained oligomers were further analyzed by ESI-MS spectra with theoretical in-source fragmentation calculations to evaluate the dispersity and potential side reactions (Figures 3e and S28–S30). Ion peaks attributable to oligomeric species with varying numbers of repeating units were observed, indicating the success of the one-pot strategy and permitting clear structural elucidation of the DP5. No peaks attributable to the monomeric product generated by water-initiated ROP (COOH– (CH_2_)_5_–OH, 132 Da) or its corresponding adduct ions were detected. Since the −O–NH_2_ end of initiator (I–O–NH_2_) initiated oligomers obtain the same *m*/*z* as I–OH-initiated oligomers, we speculate that the reduction in ligation efficiency at higher DP is related to I–O–NH_2_ initiation of polymerization at high monomer concentrations.

**Figure 3:**
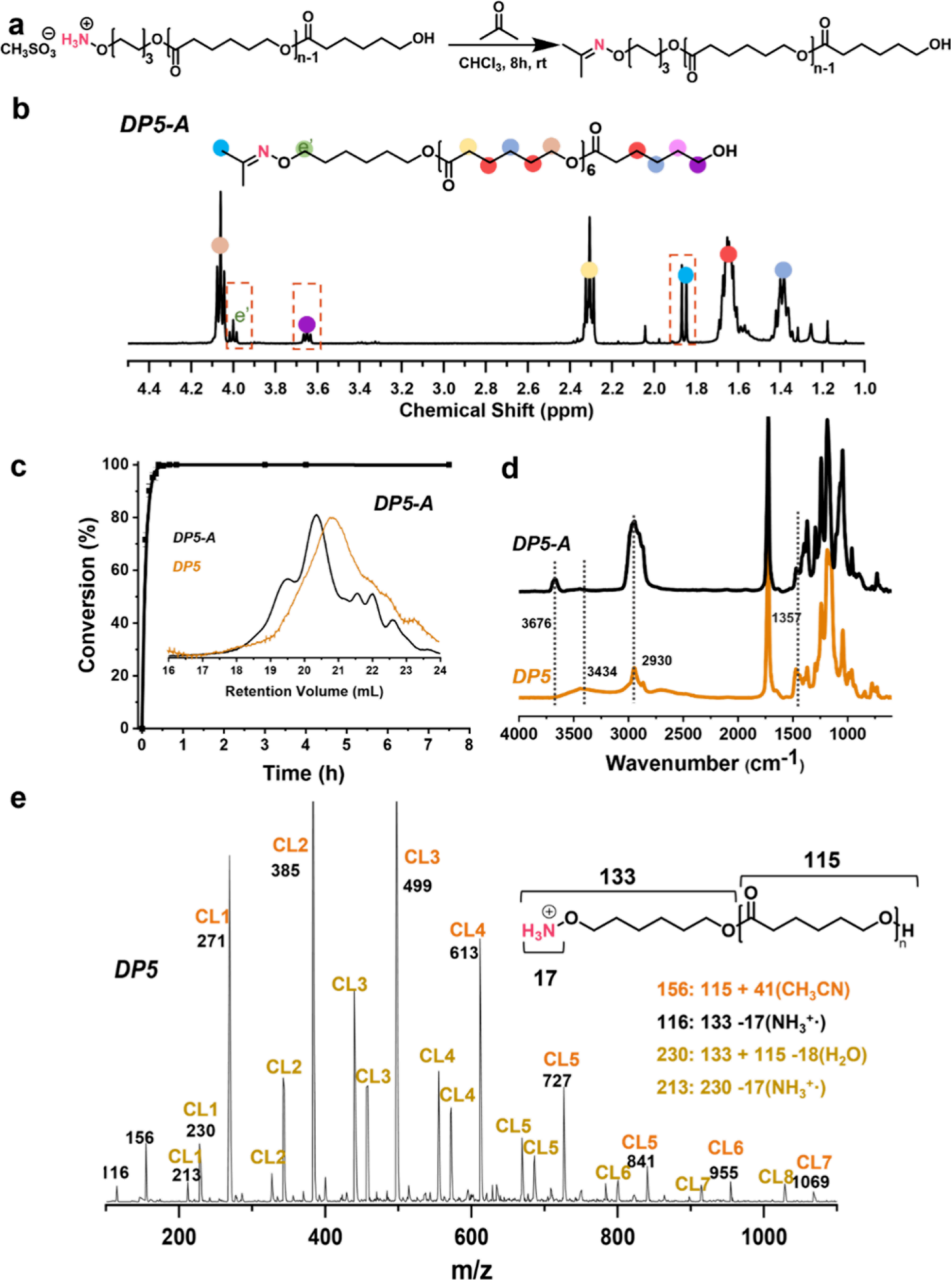
(a) Schematic representation of oxime ligation with acetone; (b) ^1^H NMR spectra of DP5-A with peak assignments; (c) kinetic plots of oxime ligation conversion: DP5-A, inset: SEC traces before and after ligation with A; (d) FTIR spectra of DP5 and ligation products with acetone; and (e) ESI-MS spectra of DP5 recorded at capillary voltage 2200 V (positive ion mode).

**Table 1:** Overview of the Different PCL Oligomers and Ligation Efficiency with Acetone

	***M***_**n**_ **(SEC, Da)**	***M***_**w**_ **(SEC, Da)**	***Đ* (SEC)**	***M***_**n**_ **(** ^**1**^ **H NMR, Da)**	**ligation efficiency (%)**
**DP5**	1100	1900	1.7	900	
**DP10**	1900	3400	1.9	2000	
**DP20**	2800	5700	1.9	2500	
**DP5-A**	1200	2800	2.4	1000	>95
**DP10-A**	3400	7200	1.9	1900	>95
**DP20-A**	3000	4300	1.4	2500	>95

Further structural assignment by HSQC, COSY, and ^13^C spectroscopy confirmed key signals on the initiator for DP5 (Figures S19–21), both before and after ligation. Because Boc deprotection proceeds through a transient *tert*-butyl cation, this intermediate can generate the side products observed at low parts per million in the ^1^H NMR spectrum (Figure 3a). After ROP, the protons of the initiator shifted upfield due to increased shielding. In the deprotected initiator, protonation of the aminooxy group caused broadening and overlap of the Boc–O–CH_2_– (*e*) signal with the PCL backbone peak at around 4.06 ppm, obscuring its precise position. However, after ligation with acetone (DP5-A), a well-resolved triplet corresponding to the NH_3_^+^–O–CH_2_– (*e*′) proton emerged downfield at 4.00 ppm, reflecting the altered environment after oxime formation. This allowed unambiguous signal assignment and accurate calculation of ROP initiation efficiency based on integral ratios between 4.00 and 3.65 ppm. Still, since in the one-pot system Boc deprotection and ε-CL ROP occur simultaneously, there are continuous changes in catalyst speciation, initiation efficiency, and chain-transfer processes. These conditions provide reliable end-group fidelity but do not support the uniform initiation or narrow dispersity associated with controlled ROP. These considerations define the intrinsic limits of structural control using this method, which primarily yield monofunctionalized aminooxy functional oligomers suitable for oxime ligation.

The acetone ligation for the different DPs was performed with 1 equiv (0.04 mmol) of acetone to the oligomer. A representative reaction of oligomer ligation with acetone is illustrated in Figure 3a. In the ^1^H NMR spectra of DP5-A (Figures 3b and S6), the protons of the methyl groups have shifted from 2.16 to 1.86 ppm upon ligation. The peak of the oligomer end-group at 3.64 ppm was chosen as the standard to calculate the ligation conversion (Figure S6) according to the ROP_I_. As shown in Table 1 and Figure S6, both DP5 and DP 10 reached ligation efficiencies exceeding 95% with acetone, although DP 20 showed a reduced ligation efficiency. Ligation of DP5 with acetone to form DP5-A also resulted in distinct changes in the FTIR spectra (Figure 3d). The broad absorption band at 3434 cm^–1^ corresponds to O–H stretching vibrations, associated with hydrogen bonding between −OH and −O–NH_3_^+^ groups (NH···OH interactions).^47,48^ In DP5-A, a sharp peak appears at 3676 cm^–1^, attributed to free −OH groups, indicating a high degree of ligation. Additionally, the intensities of the C–H stretching vibration at 2930 cm^–1^ and the C–H bending vibration at 1357 cm^–1^ became more pronounced due to the introduction of alkyl groups from the ligated acetone.^49^ These spectroscopic changes confirm the successful ligation of acetone with the oligomers in CHCl_3_, achieving over 95% conversion within 2 h at ambient temperature. ESI-MS was performed to investigate the oxime ligation with A, Figure S31. A cleavage of the N–O bond (originating from the oxime ligation) by in-source fragmentation will result in the appearance of a peak at *m*/*z* 74, attributed to the nitrilium ion (CH_3_C≡N^+^–CH_3_),^50^ demonstrating the successful ligation.

### Versatile Oxime Ligation Strategies with Diverse Aldehyde and Ketone Substrates

Further investigation of the oxime ligation reactions with different carbonyl compounds (1 equiv of either aldehyde or ketone relative to the DP5 oligomer) was conducted in CHCl_3_ at room temperature, with conversions summarized in Figure 4a. To explore substrate effects on oxime ligation, a selection of carbonyl compounds was tested: vanillin (V), acetophenone (P), benzaldehyde (B), and the bifunctional compounds terephthaldehyde (T) and 1,4-diacetylbenzene (D). All monofunctional substrates reached close to full conversion (>95%) after 8 h. Aldehydes generally display higher reactivity than ketones in nucleophilic addition reactions due to electronic and steric factors. However, no significant differences in conversion were observed after 8 h among DP5-B, DP5-P, and DP5-V (Figure 4a). To further elucidate the influence of aldehydes and ketones on oxime ligation, kinetic studies were performed. As shown in Figure 4b, all reactions exhibited a sharp increase in conversion within the first 15 min. Notably, both DP5-B and DP5-V achieved full conversion within 30 min, while acetophenone reacted much more slowly with DP5, reaching only 27 ± 2% conversion (DP5-P) at this time point. Although the final conversions after 8 h were similar for DP5-P, DP5-B, and DP5-V, the significantly slower reaction rate for DP5-P is attributed to the electron-donating methyl group in acetophenone, which reduces the electrophilicity of the carbonyl carbon. For DP5-V, the aldehyde group is conjugated to the aromatic ring (p−π conjugation), and the ring also bears hydroxyl and methoxy substituents, both electron-donating groups positioned meta and para to the aldehyde.^51^ These groups further reduce the electrophilicity of the carbonyl carbon, resulting in a slightly slower ligation rate compared to that of benzaldehyde.

**Figure 4:**
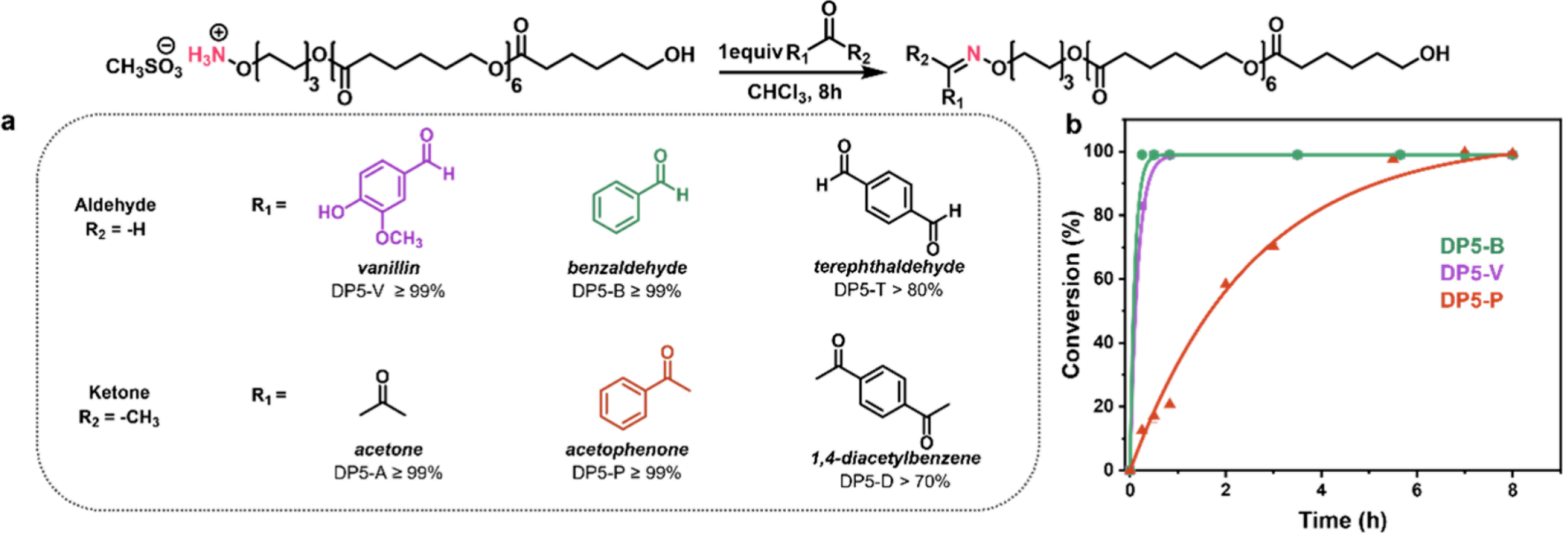
(a) Conversion rate of oxime ligation with different ketone and aldehyde substrates in organic solvents. (b) Oxime ligation conversion kinetic plots of DP5-B, DP5-P, and DP5-V.

The oxime ligation reactivity of bifunctional carbonyl compounds, 1,4-diacetylbenzene (D) and terephthalaldehyde, was investigated using a molar ratio of D or T to DP5 of 1:2, corresponding to a 1:1 stoichiometry of carbonyl to the protonated aminooxy groups. Notably, the resulting ligation products, DP5-D and DP5-T, exhibited different conversion efficiencies: >70% for DP5-D and >80% for DP5-T. Similarly, as before, the difference is attributed to the electron-donating methyl groups adjacent to the carbonyl in D, which reduce the electrophilicity of the carbonyl carbon, thereby slowing the ligation reaction compared to T. In contrast to the monofunctional analogues, the ligation reactions involving bifunctional substrates are more complex. Both D and T contain two carbonyl groups, and ^1^H NMR analysis confirmed the formation of a mixture of mono- and bis-oxime products in both cases. Importantly, neither compound achieved complete ligation, and the overall conversions remained lower than those observed for the corresponding monofunctional ligation products, DP5-P and DP5-B. We propose that the reduced ligation efficiency observed for the bifunctional substrates D and T arises from electronic effects. The meta-directing electron-withdrawing groups (−CHO and −COR) decrease overall reactivity compared with monofunctional substrates. After the first oxime forms, the oxime group donates electron density to the aromatic ring, which reduces the electrophilicity of the remaining carbonyl. As a result, the second oxime ligation proceeds more slowly. This electronic deactivation explains the accumulation of monoligated species and the lower overall conversion. The effect is more pronounced for diketone substrate D, consistent with the lower intrinsic electrophilicity of ketones relative to aldehydes.

To further extend the oxime ligation protocol, we explored the reactivity of DP5 in mixed solvent systems, including the reducing end of carbohydrates. Initial tests were conducted using D-(+)-glucose in various solvents, namely, H_2_O, DMSO, and a DMSO/H_2_O mixture. However, the limited mutual solubility of DP5 and glucose in these systems hindered the ligation process. In both H_2_O and DMSO, the mixtures appeared as suspensions, indicating inadequate solubilization of one or both components. Since sufficient solubility of both reactants is essential for effective ligation, we employed a mixed aqueous solvent system composed of H_2_O/MeOH/CHCl_3_ (1:3:1) to assess the feasibility of ligation with various aldehydes and ketones. Ligation reactions with vanillin (V_2_) and acetone (A_2_) were evaluated in this solvent system and compared to reactions in CHCl_3_. Notably, the ligation between DP5 and vanillin proceeded efficiently in the mixed solvent, reaching >93% conversion after 8 h at ambient temperature (Figure 5), comparable to the performance in pure CHCl_3_. In contrast, the reaction between DP5 and levulinic acid (L) showed a significantly lower ligation efficiency (≥70%), which can be attributed to a cyclized side product in the reaction.

**Figure 5:**
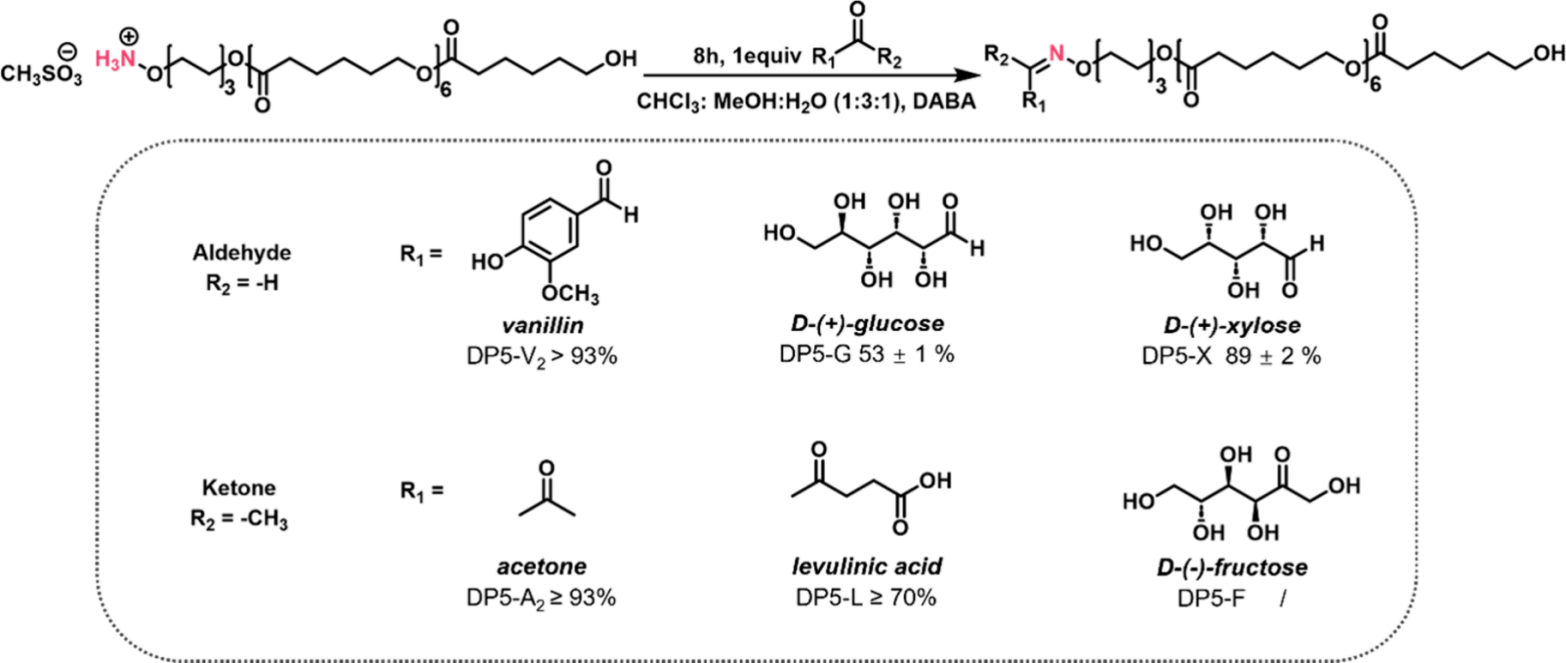
Conversion of DP5 oxime ligation with different ketone and aldehyde substrates in mixed aqueous solvents.

When performing ligation at the reducing end of carbohydrates, it is crucial to consider the mutarotation equilibrium that occurs in aqueous solution.^52^ This equilibrium involves interconversion between the cyclic (closed-ring) forms and the open-chain aldehyde form, which can also extend to the ligated products. The resulting sugar oximes exist in a complex tautomeric equilibrium comprising the anti-(Z) and syn-(E) acyclic isomers as well as the α- and β-cyclic forms. Consequently, the ligation products in our mixed aqueous system are expected to undergo interconversion among these various forms. To quantify the ligation efficiency, we calculated the total conversion based on the sum of the integrals of the anomeric hydrogen signals in the ^1^H NMR spectra. D-(+)-Glucose and D-(+)-xylose were selected as representative carbohydrate models due to their stability as the most common hexose and pentose sugars, respectively.^53^ In Figures S15 and S16, the ^1^H NMR spectra display peaks at 7.32 (E), 6.69 (Z), and 5.19 ppm (β), confirming the presence of multiple tautomeric forms of the sugar oxime ligation products for both glucose and xylose. As shown in Figure 5, xylose demonstrated an excellent ligation efficiency with DP5 (DP5-X). Compared to glucose, xylose exhibits a significantly higher proportion of its aldehyde form, approximately ten times greater, in aqueous solution.^54,55^ This higher availability of the reactive carbonyl group correlates well with the observed order of conversion presented in Figure 5 (DP5-X 89 ± 2%, DP5-G 53 ± 1%). In addition, D-fructose, a ketose and structural isomer of the aldose d-glucose, was selected to investigate the influence of the carbonyl structure on the oxime ligation efficiency. The reaction between fructose and DP5 was evaluated by ^1^H NMR spectroscopy. Compared to glucose, fructose exhibits a slightly higher proportion of its keto tautomeric form in D_2_O (∼0.50%)^55,56^ However, here no distinct NMR signals corresponding to H_6_, indicative of successful ligation, were observed; for more insights, see Scheme 1, Supporting Information. The apparent lack of reactivity of fructose under our reaction conditions may be due to the low electrophilicity of the ketone group and the influence of the aqueous reaction environment. These results highlight the potential for achieving selective ligation of carbohydrates within complex mixtures, by exploiting differences in mutarotation and carbonyl reactivity in aqueous systems, favoring aldehyde-containing sugars with a higher degree of aldehydes in equilibrium.

## Conclusions

In this work, we established a one-pot synthetic strategy that combines the ROP of ε-caprolactone with in situ Boc-deprotection to generate highly reactive aminooxy-terminated PCL oligomers. The method enables simultaneous polymer growth and end-group activation, producing “click-ready” degradable oligomers directly from a single reaction setup. Here, methanesulfonic acid was identified as both enabling Boc-deprotection and catalyzing polymerization, affording functionalized oligomers with different degrees of polymerization. The resulting NH_3_^+^–O–PCL species displayed rapid and efficient oxime ligation with a broad range of aldehydes and ketones under mild conditions, achieving high conversions even in mixed aqueous media. The study revealed clear structure–reactivity relationships governed by carbonyl electrophilicity and steric effects and demonstrated successful conjugation with reducing sugars such as glucose and xylose. Overall, this work delivers a robust and water-tolerant platform for introducing bio-orthogonal functionality into degradable polymers. By enabling direct, selective coupling in the presence of water, the strategy opens new possibilities for constructing sustainable polymer–biopolymer hybrids and reactive interfaces relevant to biomedical and green-materials design.

## Supplementary Material



## Notes

Additional NMR spectra, SEC traces, ESI-MS data, and supplementary figures and tables (PDF)
