# Supplementary material for: Synthesis of Click-Ready Aminooxy-Terminated Poly(ε-caprolactone) Oligomers for Oxime Ligation
Source: Bioconjug Chem. 2026 Apr 23;37(5):1005–14. doi: 10.1021/acs.bioconjchem.6c00091 (PMC13195576; doi:10.1021/acs.bioconjchem.6c00091)
Supplement: Supplementary file 1 [file bc6c00091_si_001.pdf]

Synthesis of Click-Ready Aminooxy-Terminated Poly(ε-caprolactone) Oligomers for Oxime Ligation

Weilin Zhang[a], *Karin Odelius**[a], *Peter Olsén**[b]

[a] *Wallenberg Wood Science Center, Department of Fibre and Polymer Technology, KTH Royal Institute of Technology, Stockholm 10044, Sweden*

[b]*Wallenberg Wood Science Center, Laboratory of Organic Electronics, Linköping University, Norrköping 60174, Sweden*

Supplementary Material

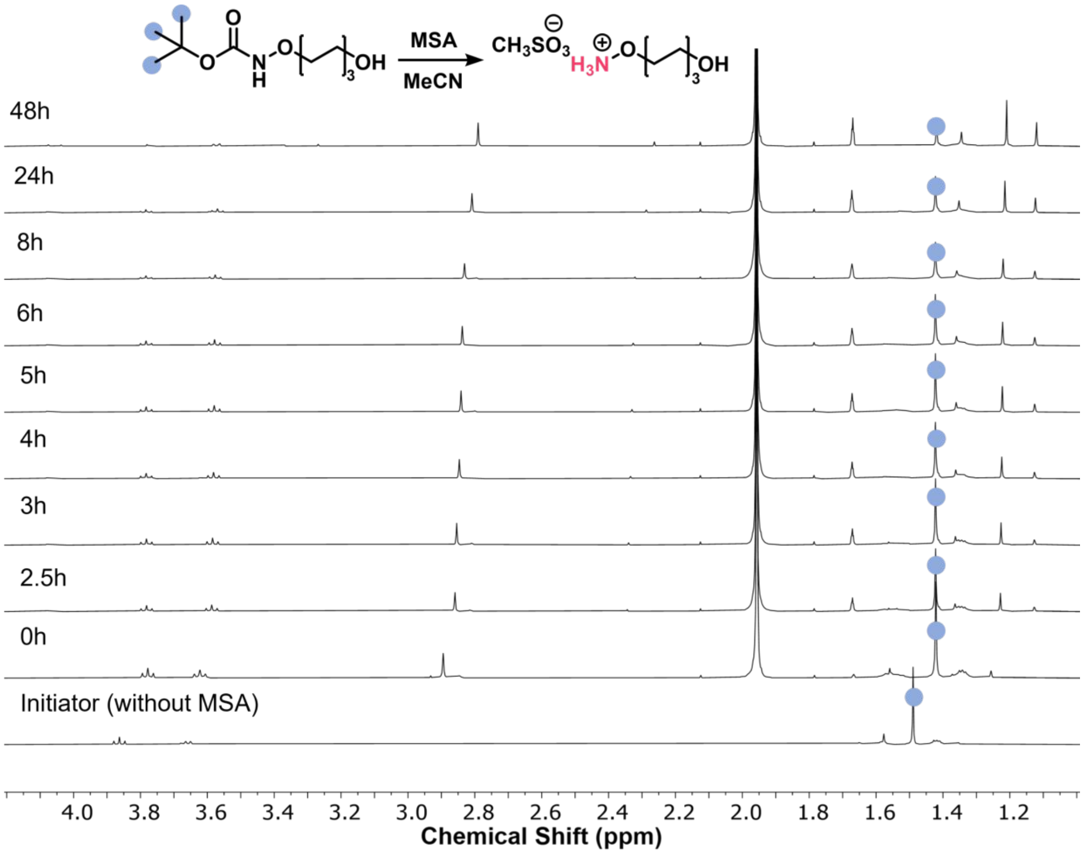

Figure S1. ^{1}H NMR spectra (400MHz) of the Boc (tert-butyloxycarbonyl group) deprotection in $CDCl_3$.

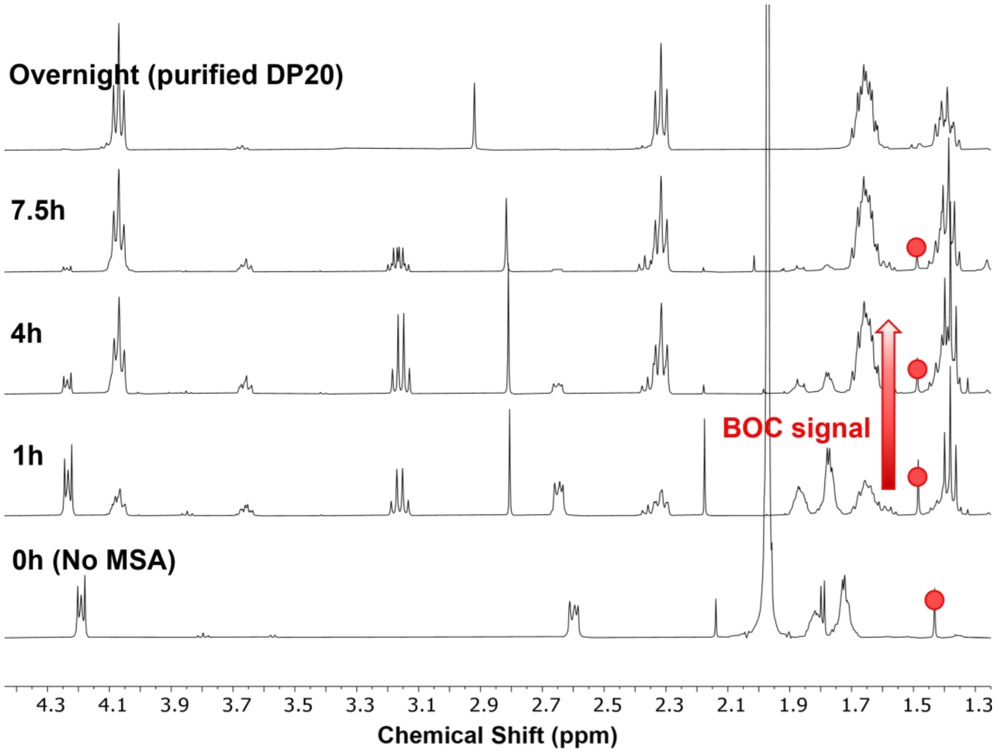

Figure S2. ^{1}H NMR spectra (400MHz) of the Boc (tert-butyloxycarbonyl group) deprotection by 3 equiv. MSA in one-pot reaction over time. Peak g (δ = 1.49 ppm) decreased over the reaction time.

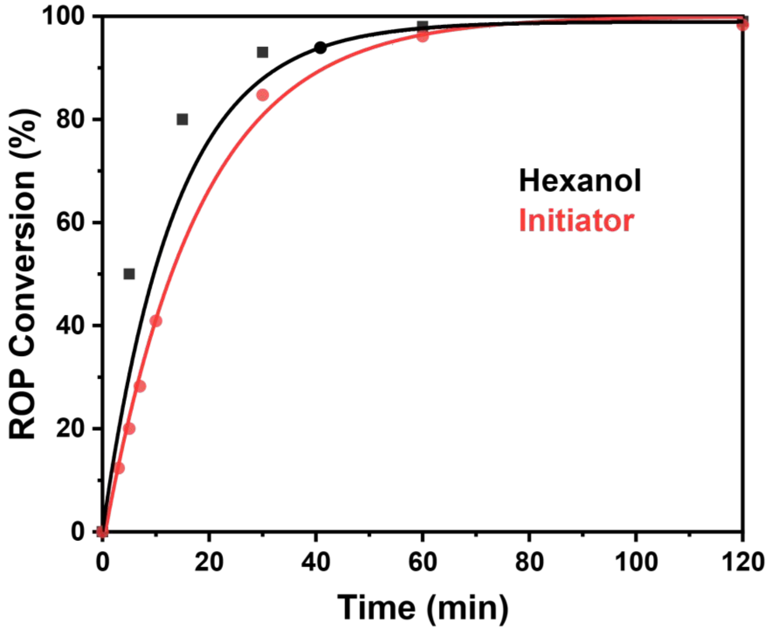

Figure S3. Plot of the conversion as a function of time for DP5 synthesis with 3 equiv MSA in MeCN at room temperature. Black: ROP initiated by hexanol; Red: ROP initiated by initiator in one-pot synthesis.

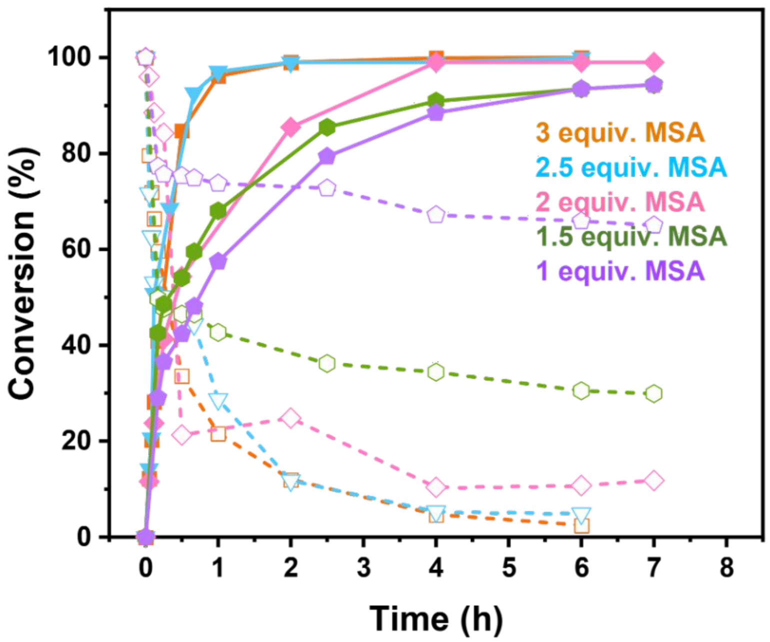

Figure S4. Plot of the conversion as a function of time of the one-pot strategy for ring-opening polymerization and deprotection in MeCN at room temperature, with varying MSA molar ratios ranging from 1 to 3 equivalents. Solid line: ROP, dashed line: Boc deprotection.

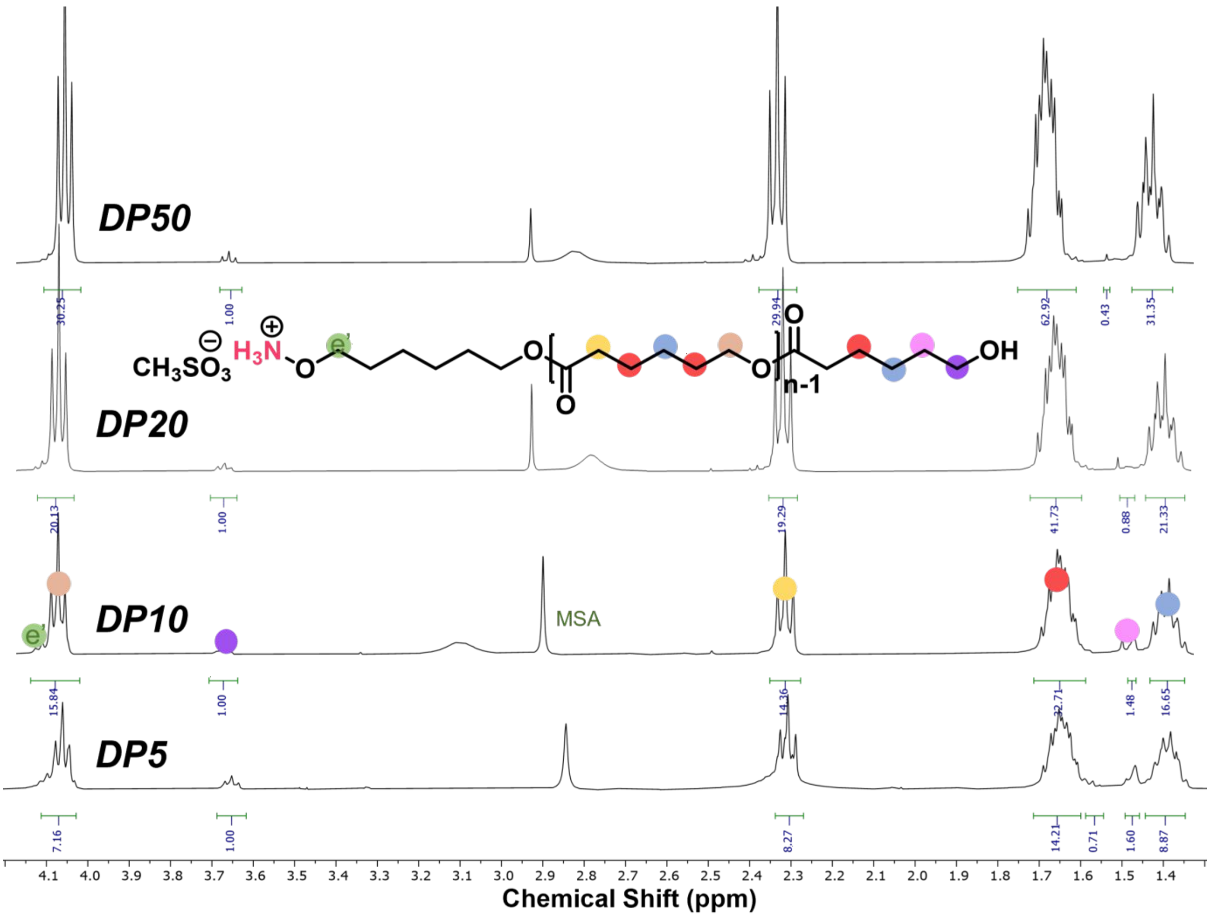

Figure S5. ^{1}H NMR spectra (400MHz) of DP5, DP10, DP20 and DP50 in $CDCl_3$.

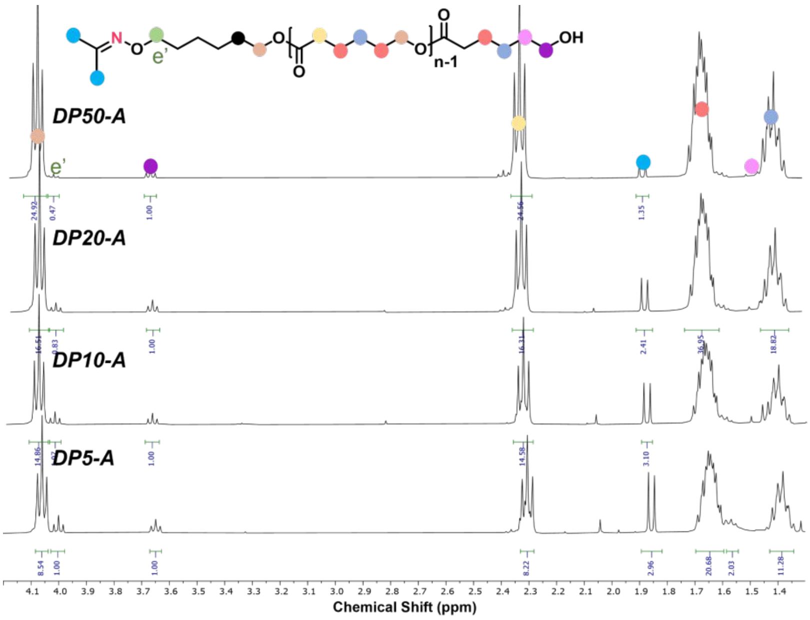

DP50-A

$$\mathrm{ROP_I}(\%) = \frac{I_{4.00}}{I_{3.65}} \cdot 100 = 47\%$$

$$\text{Conversion } (\%) = \frac{I_{1.86}}{3 \cdot I_{4.00}} \cdot 100 = 98\%$$

DP20-A

$$\mathrm{ROP_I}(\%) = \frac{I_{4.00}}{I_{3.65}} \cdot 100 = 83\%$$

$$\text{Conversion } (\%) = \frac{I_{1.86}}{3 \cdot I_{4.00}} \cdot 100 = 97\%$$

DP10-A

$$\mathrm{ROP_I}(\%) = \frac{I_{4.00}}{I_{3.65}} \cdot 100 = 100\%$$

$$\text{Conversion } (\%) = \frac{I_{1.86}}{3 \cdot I_{4.00}} \cdot 100 = 100\%$$

DP5-A

$$\mathrm{ROP_I}(\%) = \frac{I_{4.00}}{I_{3.65}} \cdot 100 = 100\%$$

$$\text{Conversion } (\%) = \frac{I_{1.86}}{3 \cdot I_{4.00}} \cdot 100 = 100\%$$

Figure S6. ROP conversion and ^{1}H NMR spectra (400MHz) of DP5-A, DP10-A, DP20-A and DP50-A in $CDCl_3$.

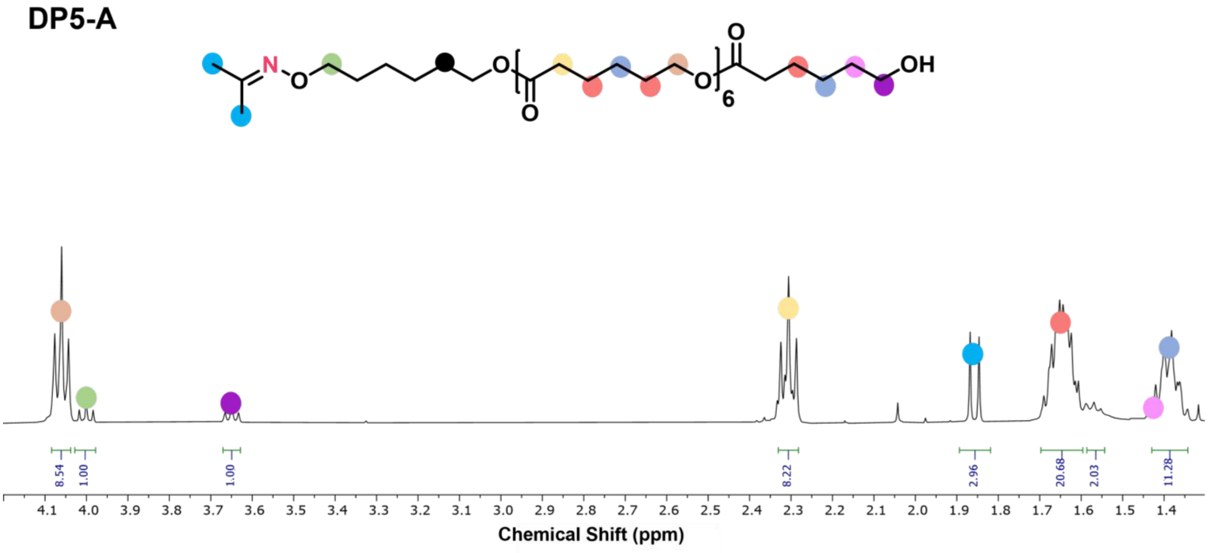

Figure S7. ^{1}H NMR spectra (400MHz) of DP5-A in $CDCl_3$.

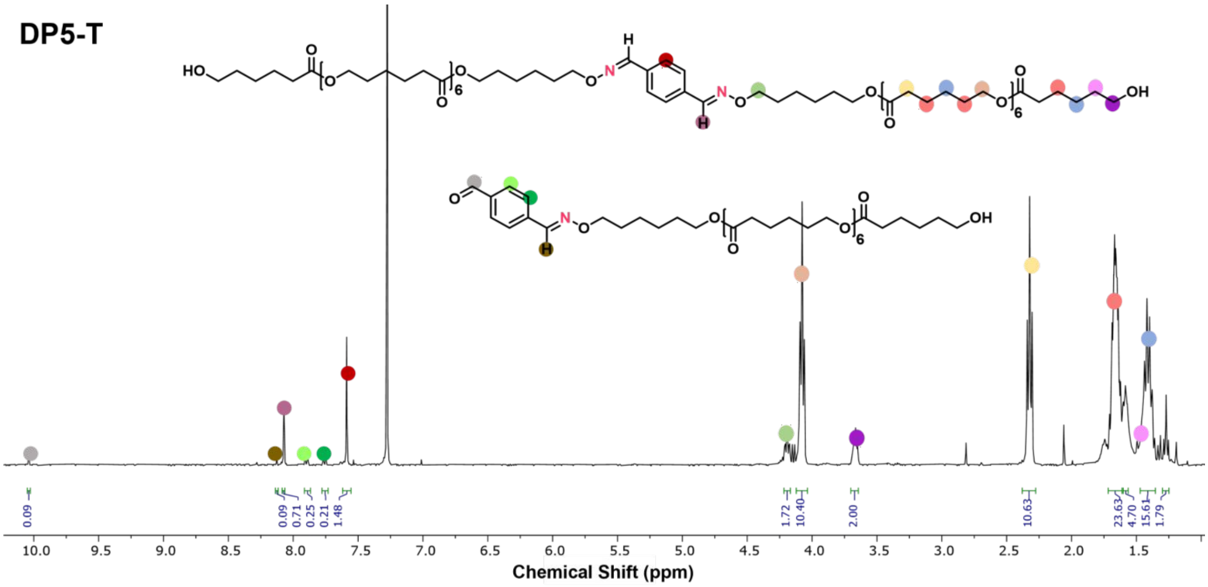

Figure S8. ^{1}H NMR spectra (400MHz) of DP5-T in $CDCl_3$.

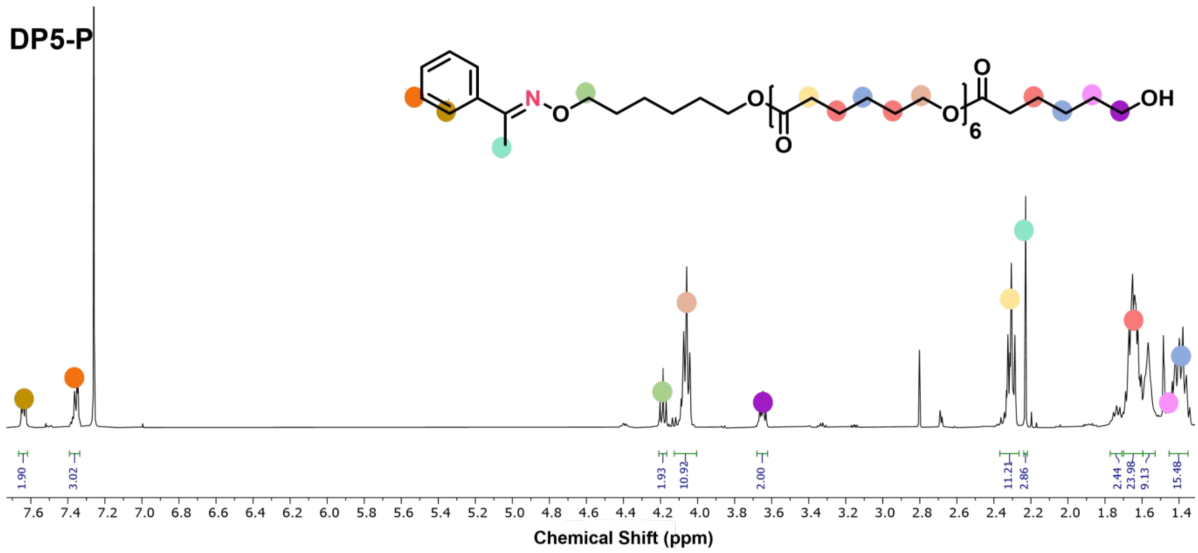

Figure S9. ^{1}H NMR spectra (400MHz) of DP5-P in $CDCl_3$.

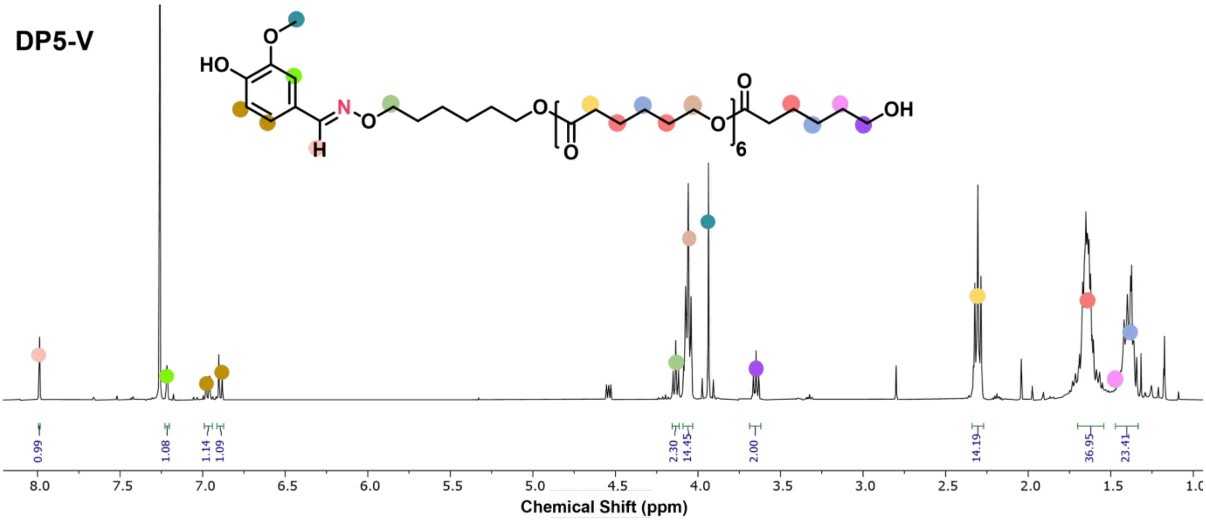

Figure S10. ^{1}H NMR spectra (400MHz) of DP5-V in $CDCl_3$.

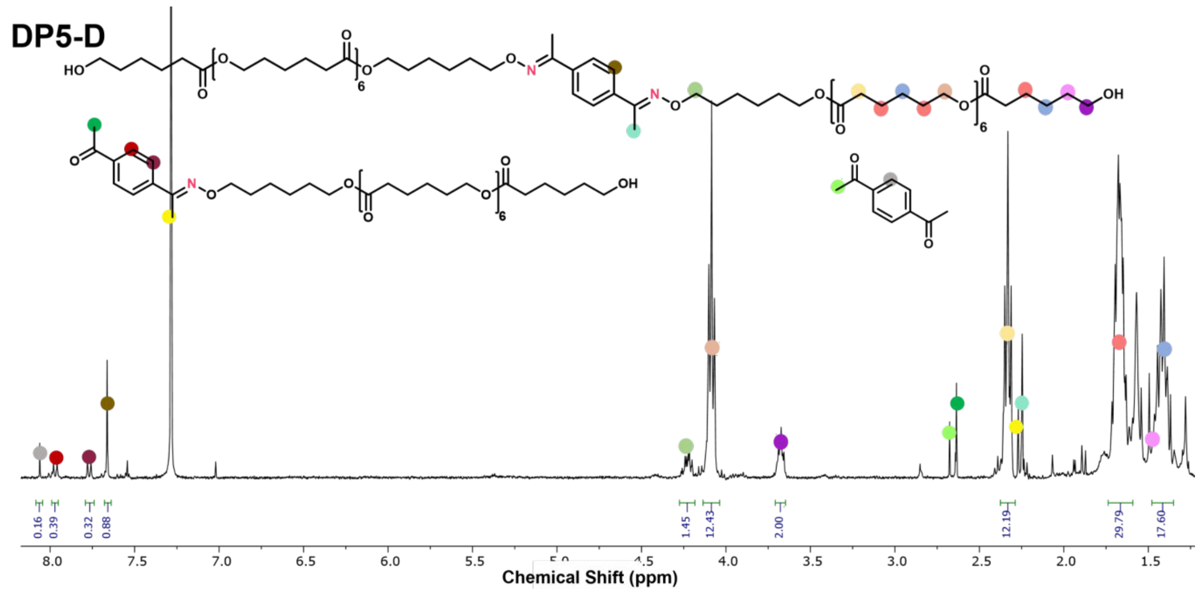

Figure S11. ^{1}H NMR spectra (400MHz) of DP5-D in $CDCl_3$.

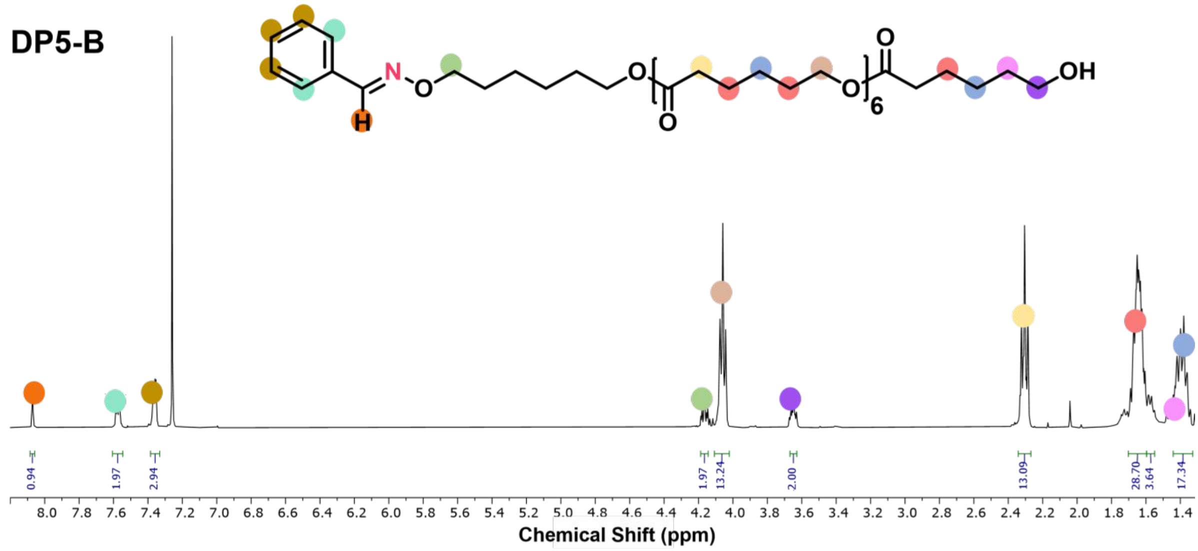

Figure S12. ^{1}H NMR spectra (400MHz) of DP5-B in $CDCl_3$.

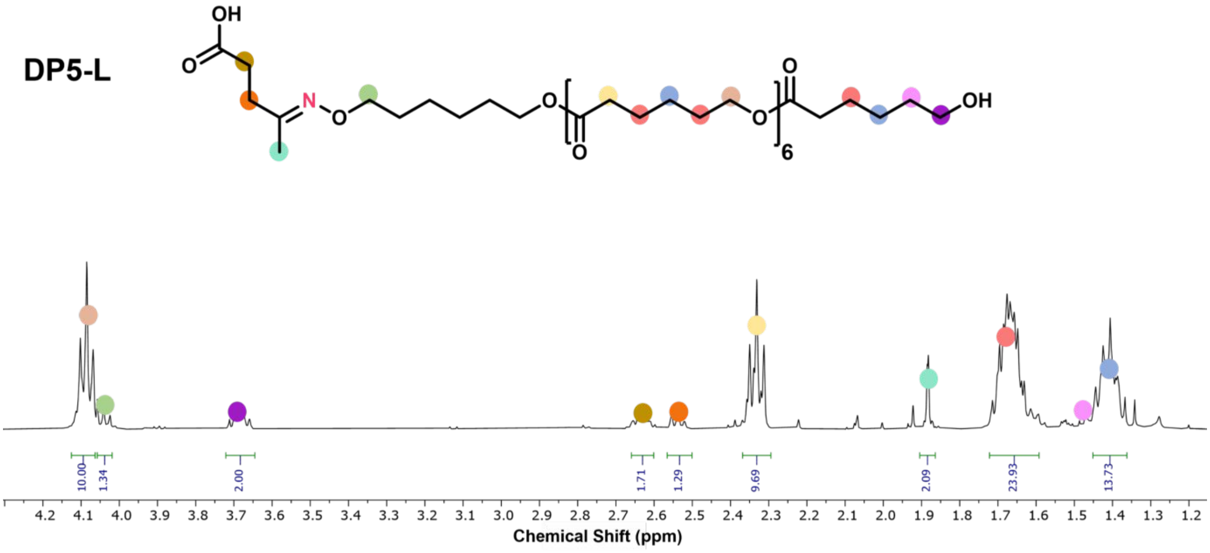

Figure S13. ^{1}H NMR spectra (400MHz) of DP5-L in $CDCl_3$.

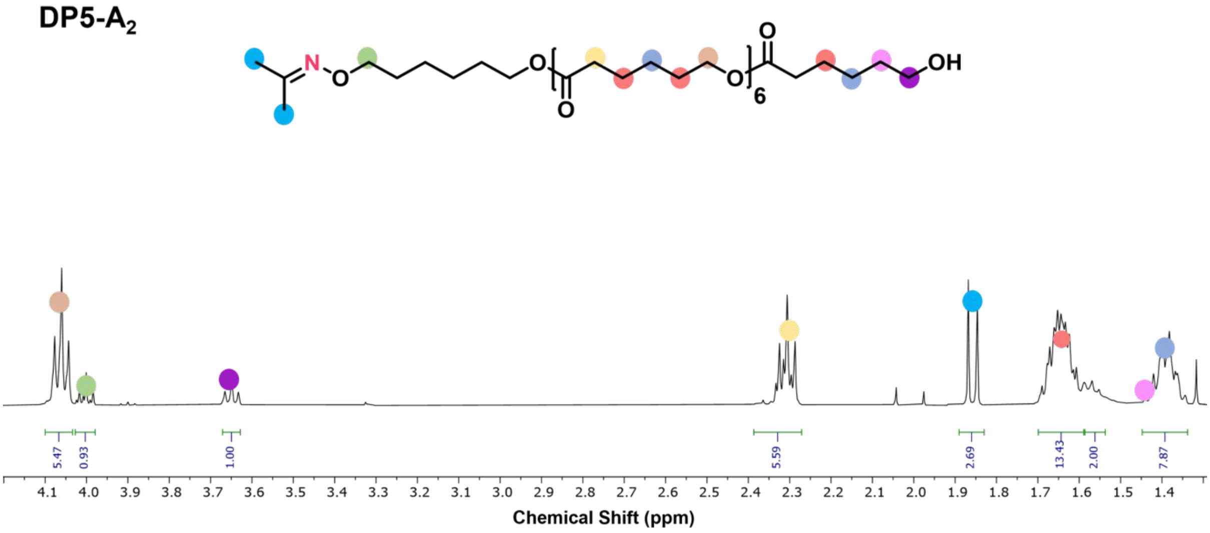

Figure S14. ^{1}H NMR spectra (400MHz) of DP5-A_2 in $CDCl_3$.

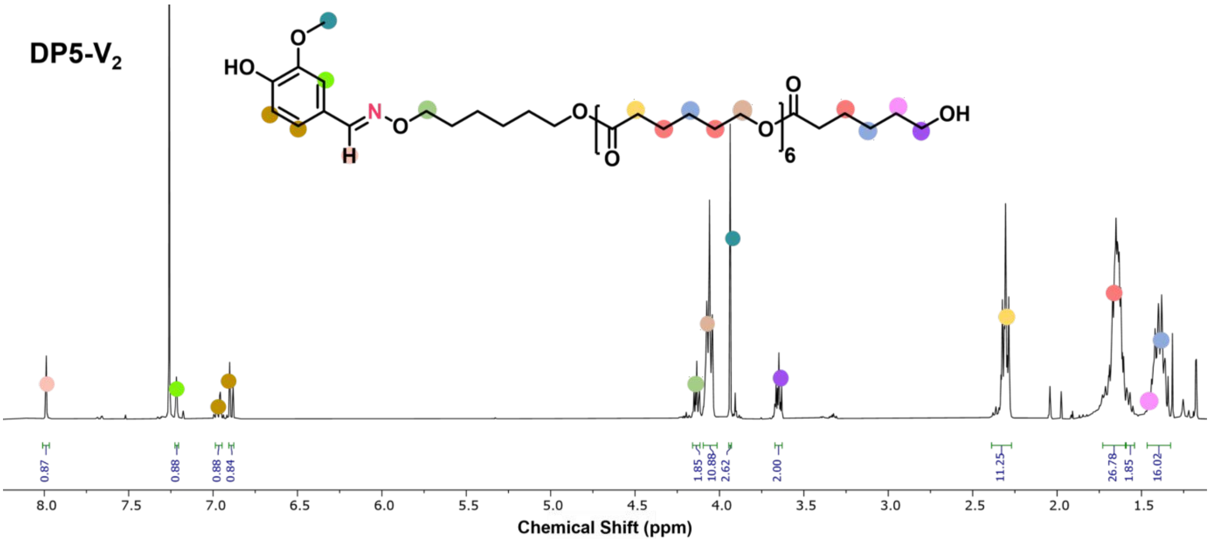

Figure S15. ^{1}H NMR spectra (400MHz) of DP5-V_2 in $CDCl_3$.

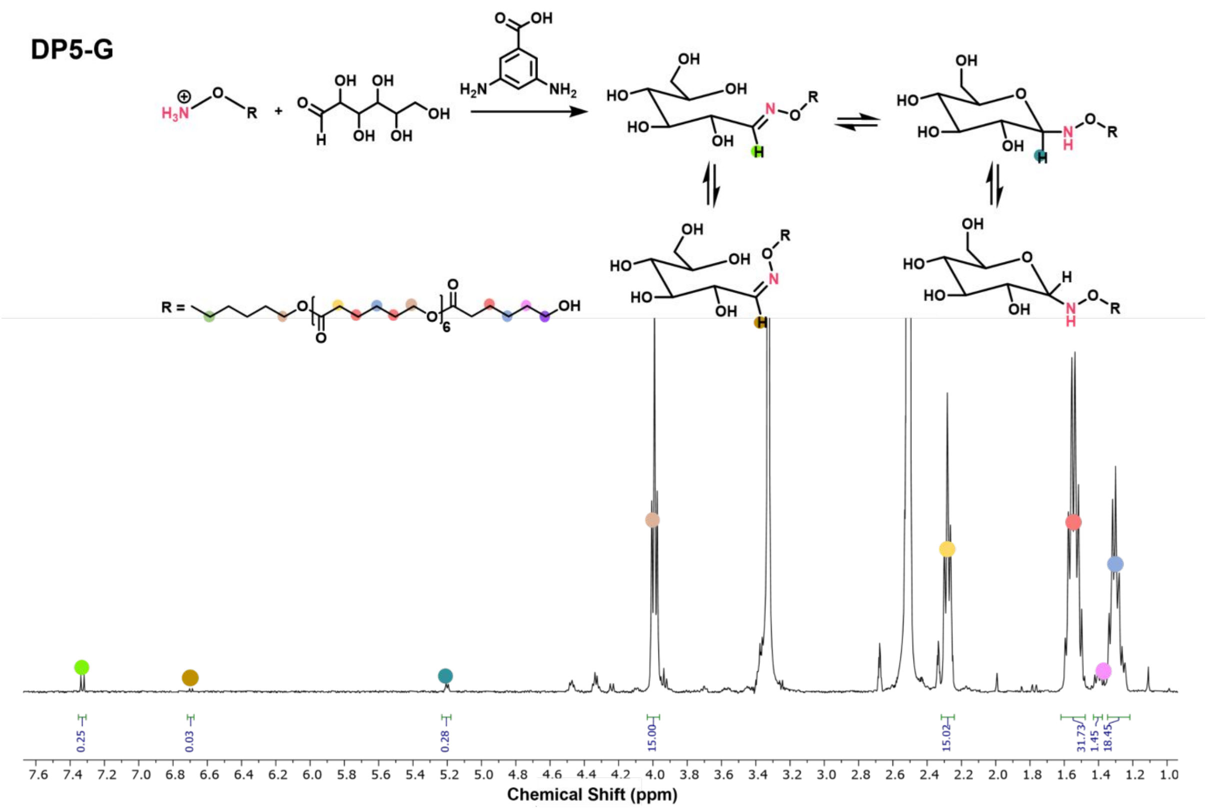

Figure S16. ^{1}H NMR spectra (400MHz) of DP5-G in DMSO-d_6.

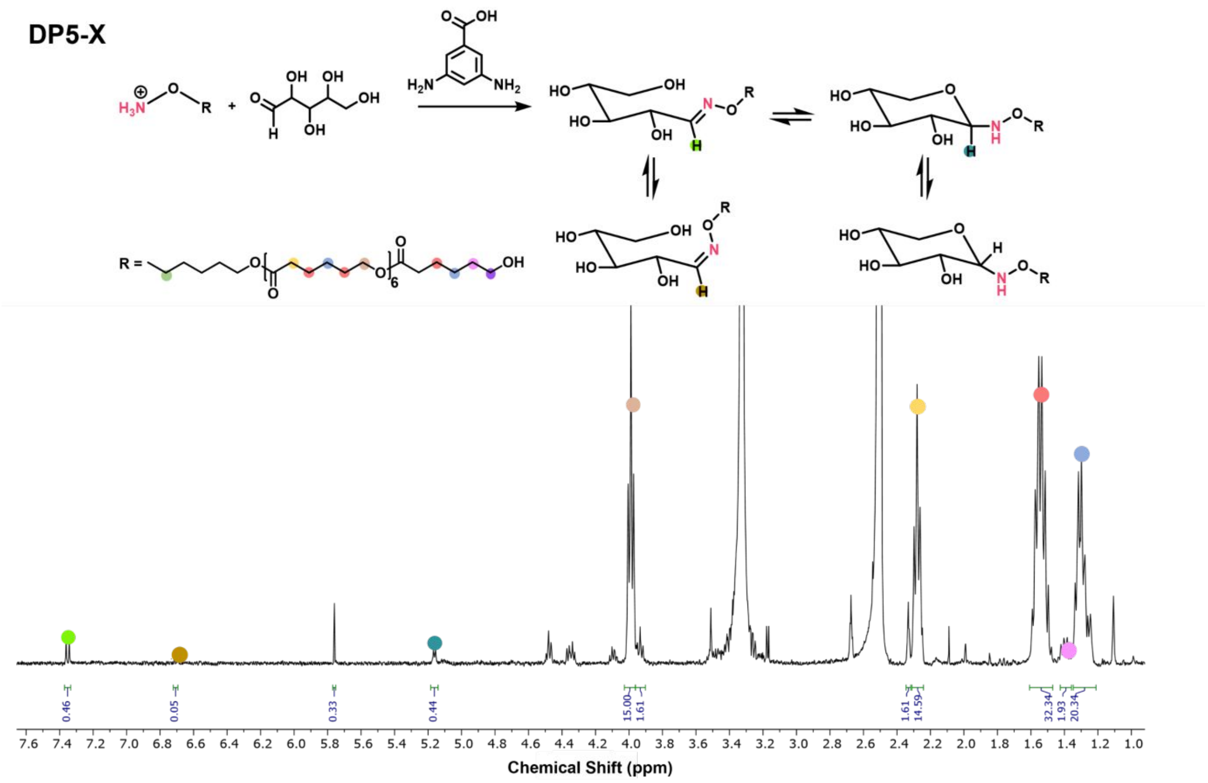

Figure S17. ^{1}H NMR spectra (400MHz) of DP5-X in DMSO-d_6.

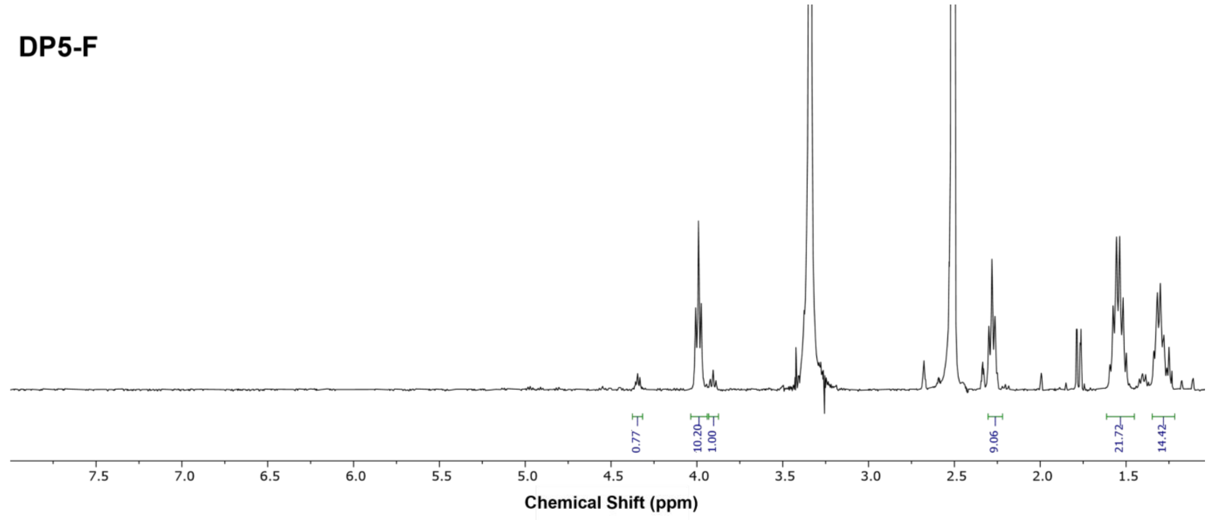

Figure S18. ^{1}H NMR spectra (400MHz) of DP5-F in DMSO-d_6.

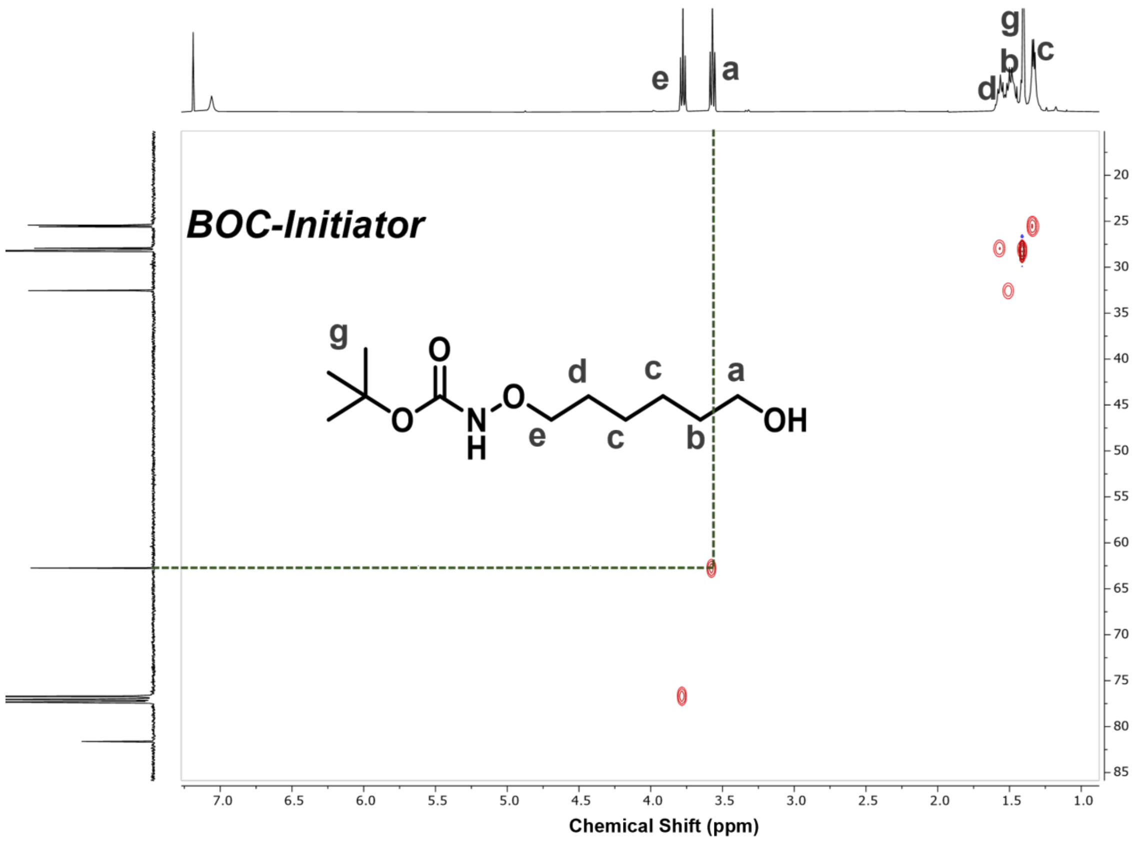

Figure S19. [^{1}H, ^{13}C]-HSQC spectra (400MHz) of tert-butyloxycarbonyl-6-aminoxyl-1-hexanol in $CDCl_3$.

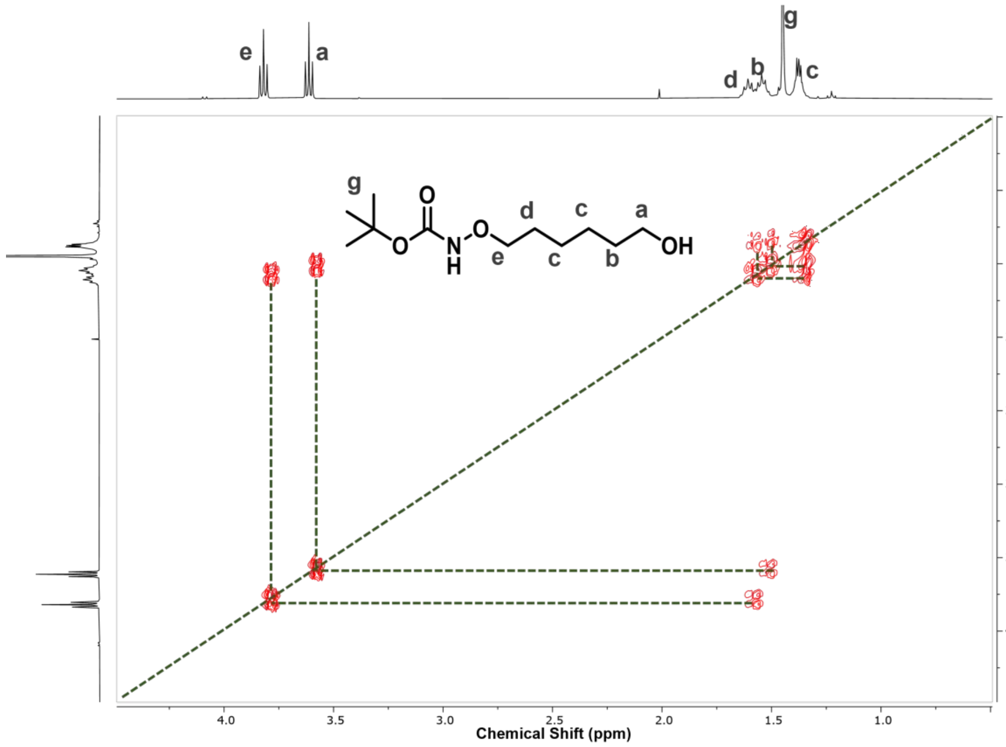

Figure S20. [^{1}H, ^{1}H]-COSY spectra (400MHz) of tert-butyloxycarbonyl-6-aminoxyl-1-hexanol in $CDCl_3$.

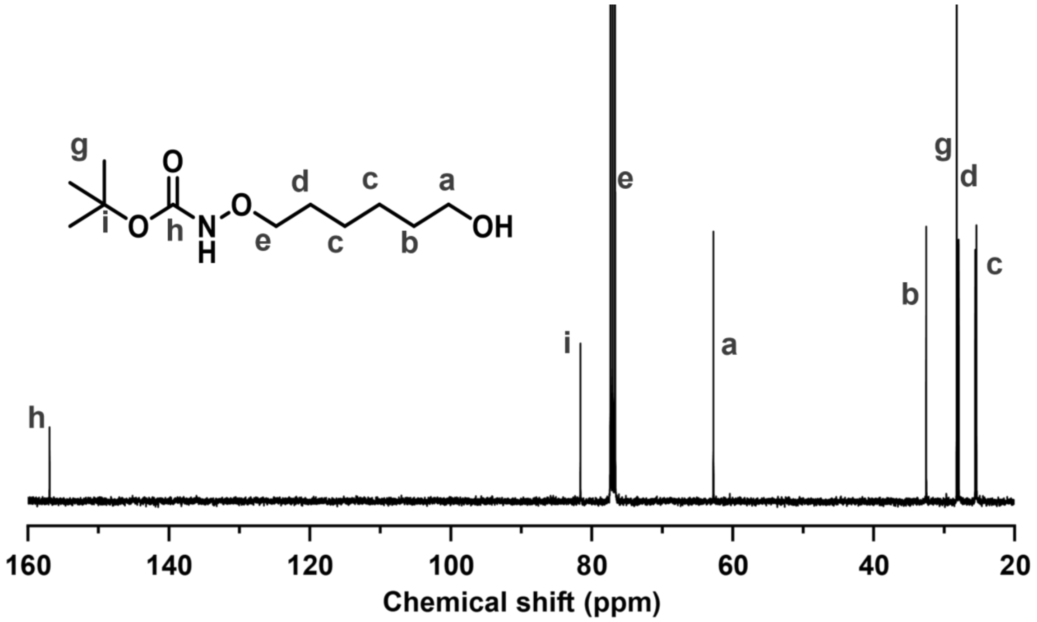

Figure S21. ^{13}C spectra (400MHz) of tert-butyloxycarbonyl-6-aminoxyl-1-hexanol in $CDCl_3$.

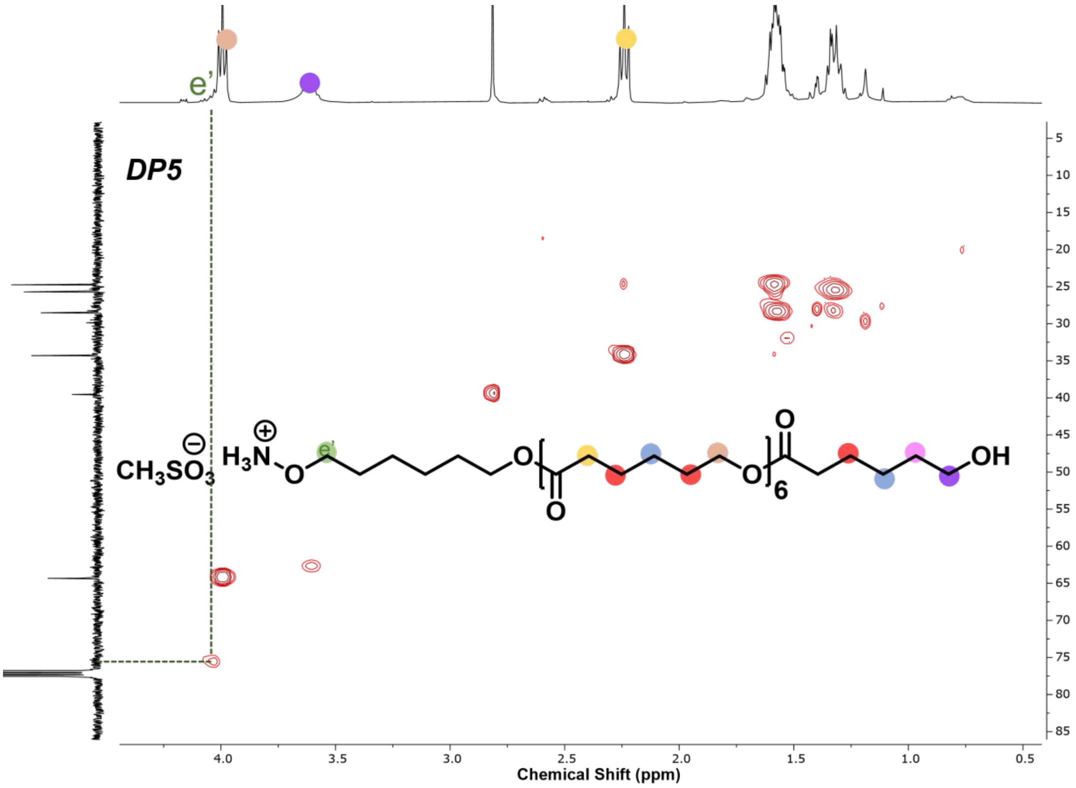

Figure S22. [^1H, ^{13}C]-HSQC spectra (400MHz) of DP5 in $CDCl_3$.

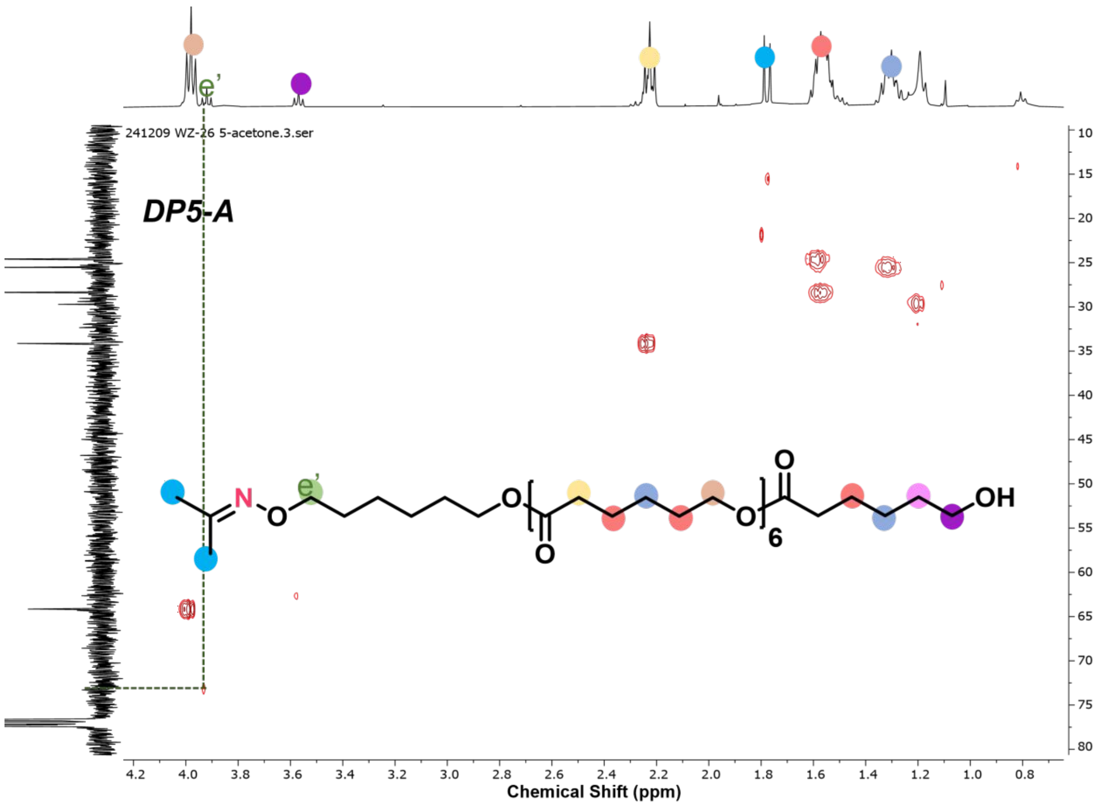

Figure S23. [^{1}H, ^{13}C]-HSQC spectra (400MHz) of DP5-A in $CDCl_3$.

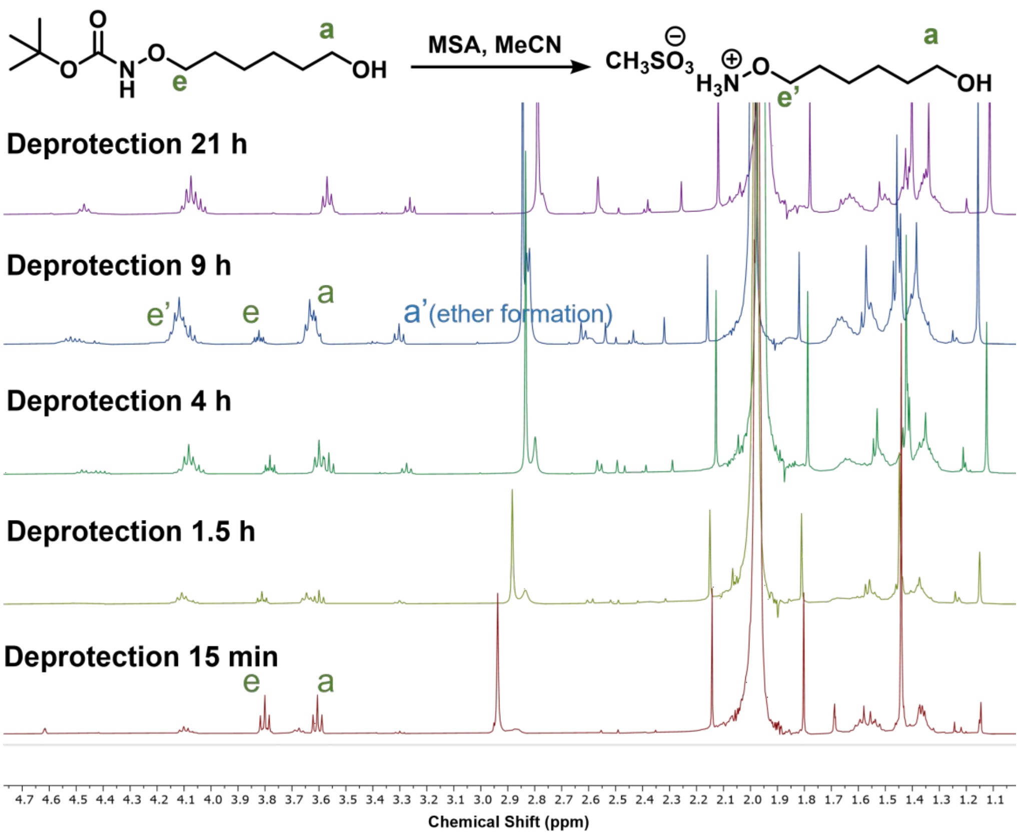

Figure S24. ^{1}H NMR spectra of Boc-deprotection over time at 2 equiv. MSA in $CDCl_3$.

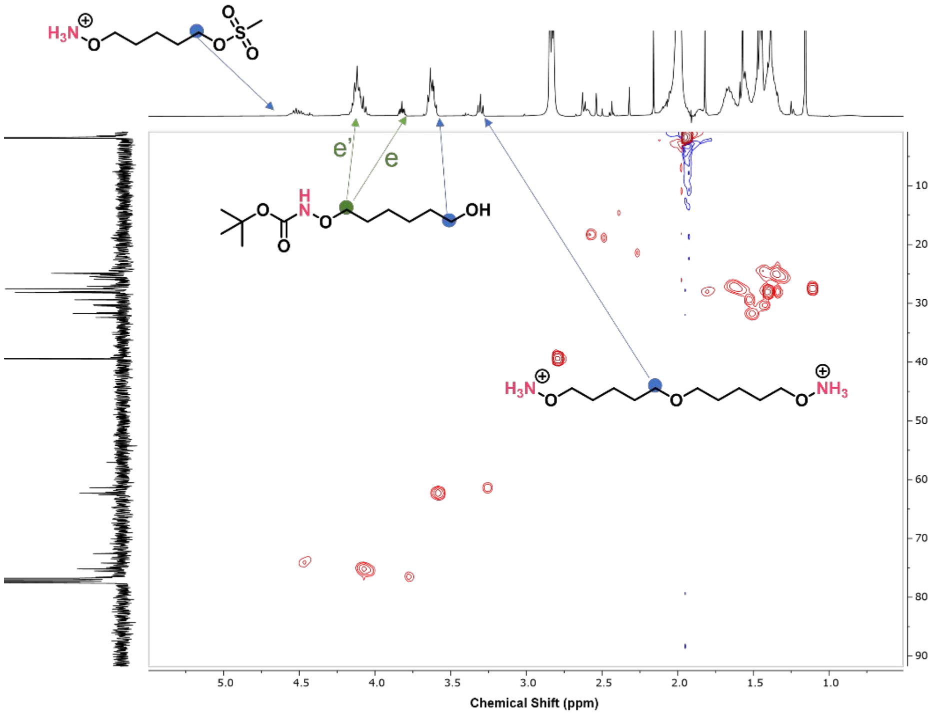

Figure 25. [^{1}H, ^{13}C]-HSQC spectra (400MHz) of Boc-deprotection at 9 h with 2 equiv. MSA in $CDCl_3$.

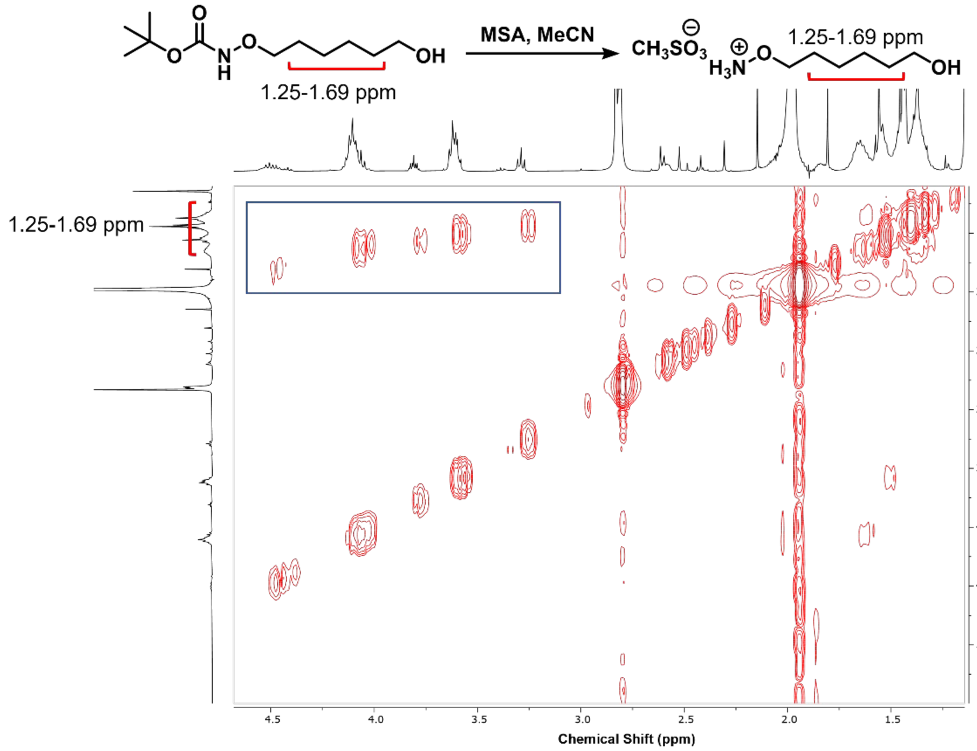

Figure 26. [^{1}H, ^{1}H]-COSY spectra (400MHz) of Boc-deprotection at 9 h with 2 equiv. MSA in $CDCl_3$.

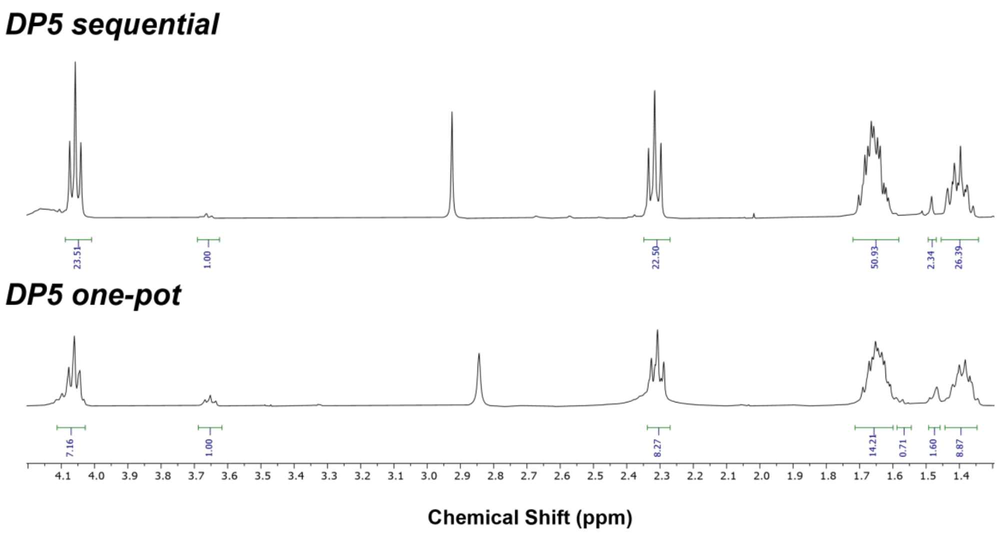

Figure S27. ^{1}H NMR spectra of DP5 from the one-pot synthesis and DP5 from the sequential reactions in $CDCl_3$. DP5$_{sequential}$: deprotection then ROP; DP5$_{one\text{-}pot}$: deprotection and ROP simultaneously. Both reactions were run with 3 equiv. MSA in the MeCN at room temperature.

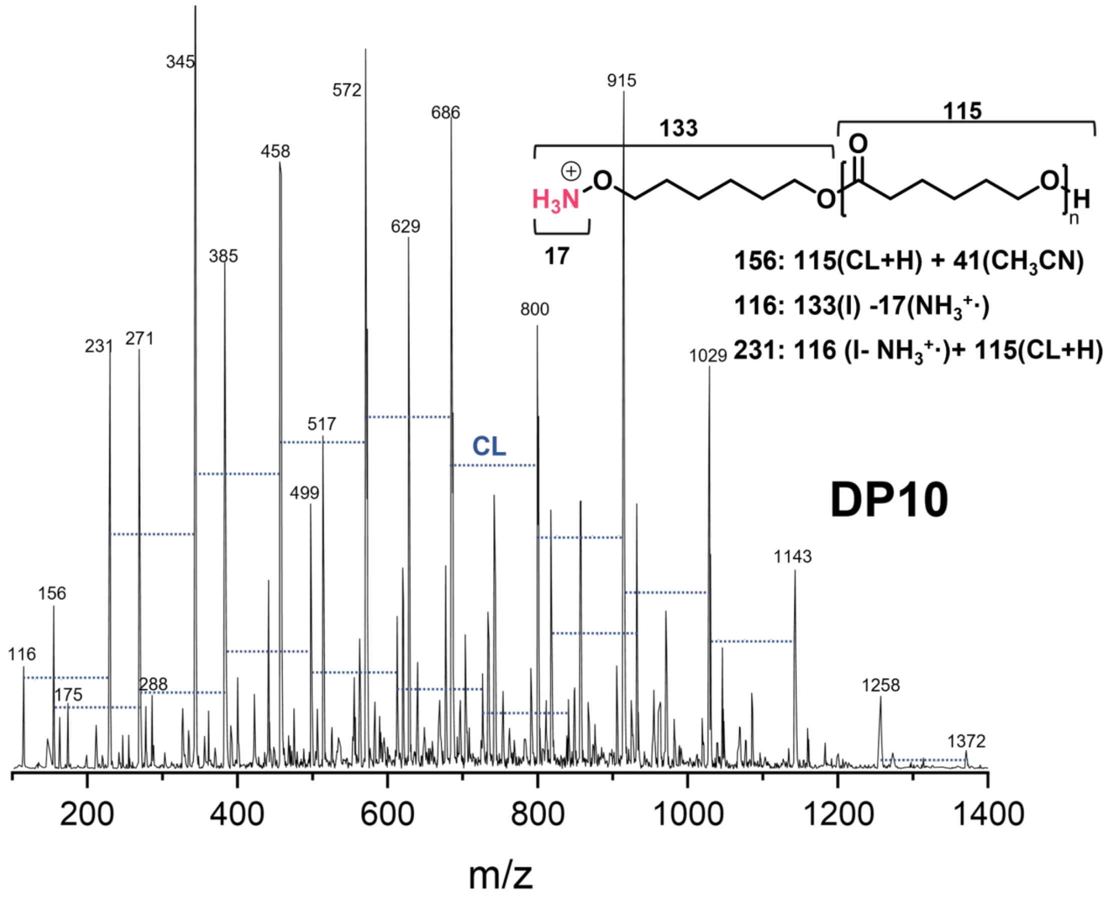

Figure S28. ESI-MS spectra of DP10 recorded at capillary voltage 2200V (positive ion mode).

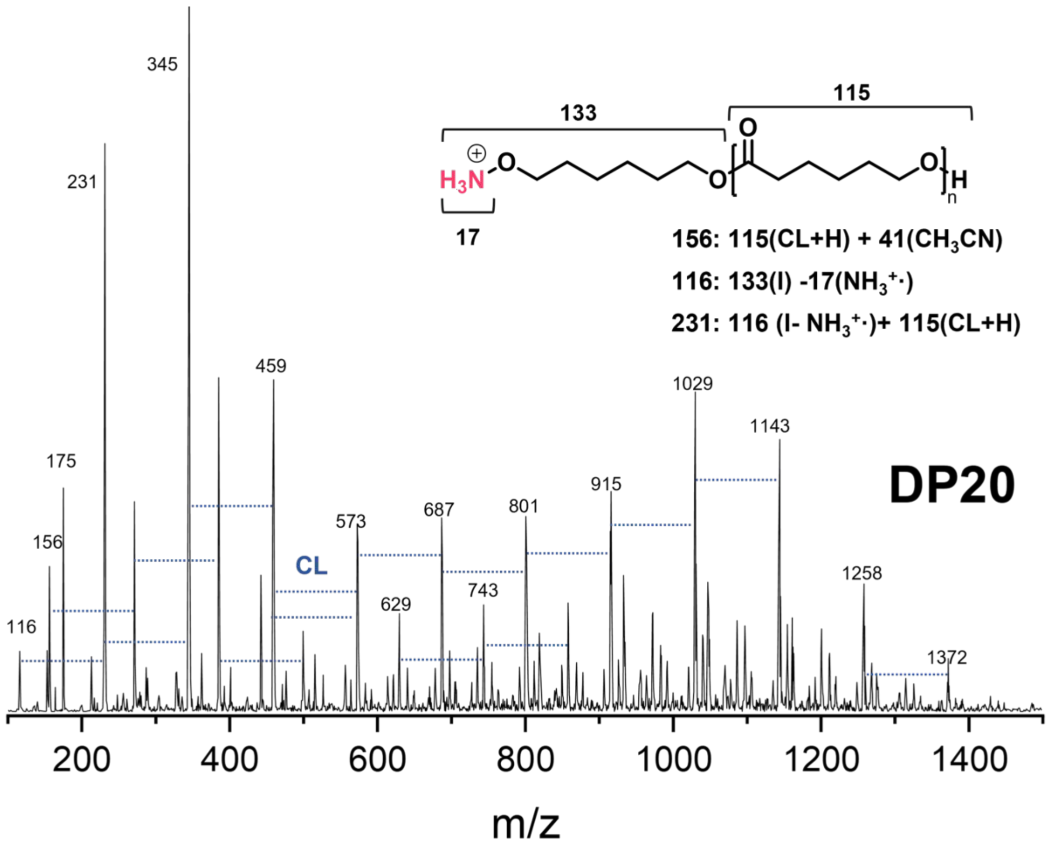

Figure S29. ESI-MS spectra of DP20 recorded at capillary voltage 2200V (positive ion mode).

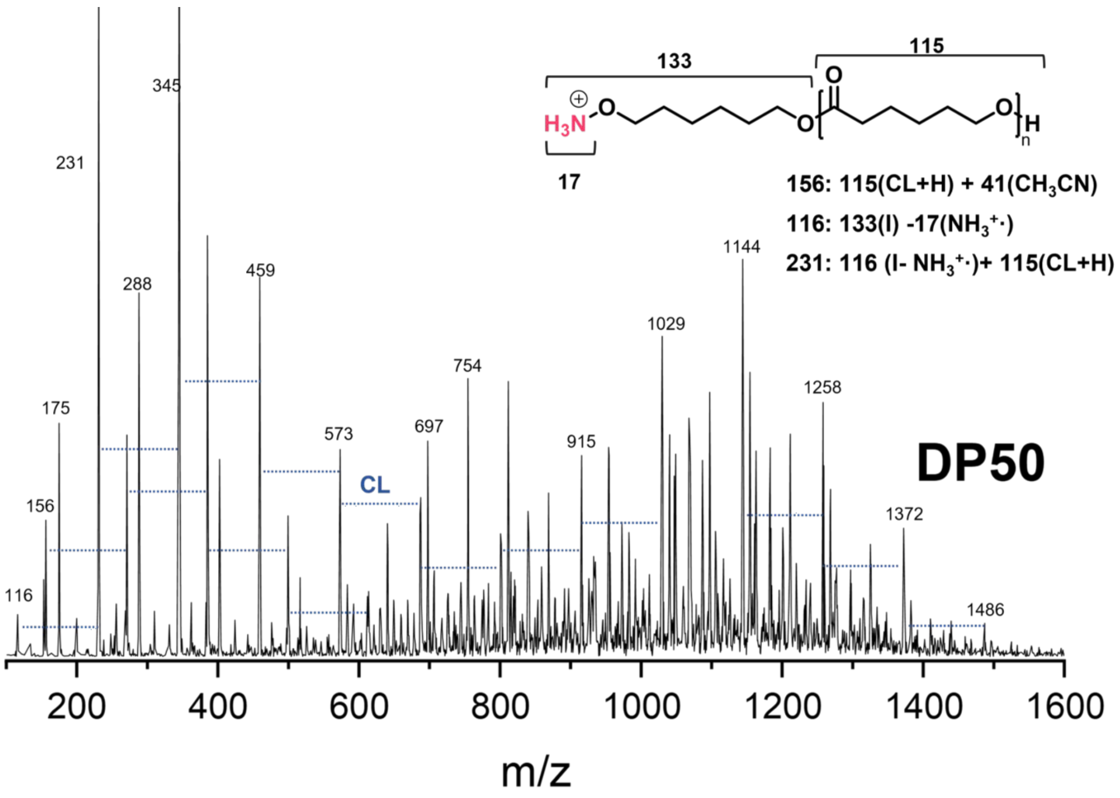

Figure S30. ESI-MS spectra of DP50 recorded at capillary voltage 2200V (positive ion mode).

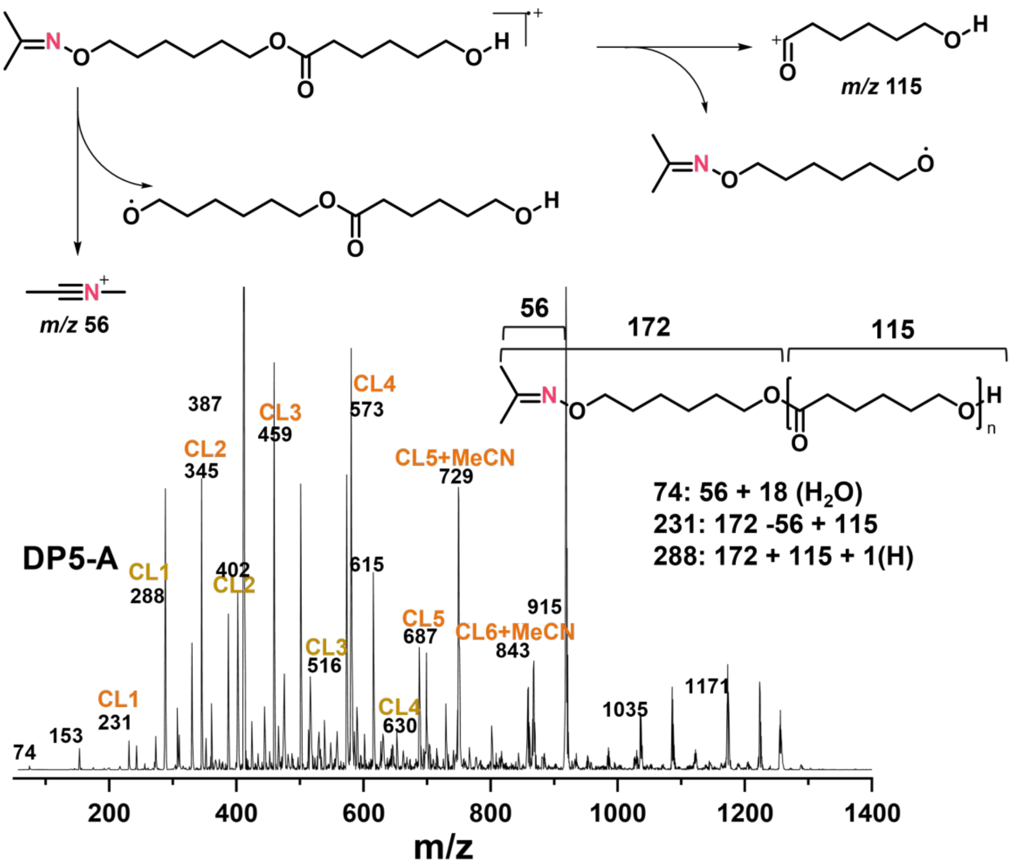

Figure S31. ESI-MS spectra of DP5-A recorded at capillary voltage 2200V (positive ion mode).

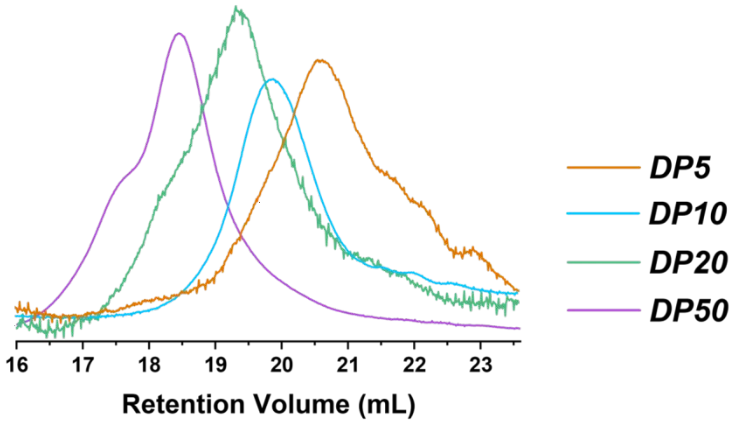

Figure S32. SEC traces of DP5, DP10, DP20 and DP50.

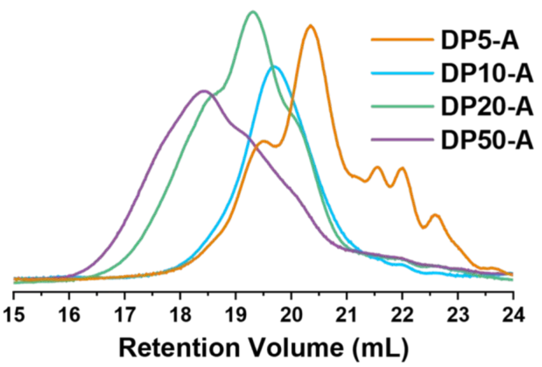

Figure S33. SEC traces of DP5-A, DP10-A, DP20-A and DP50-A.

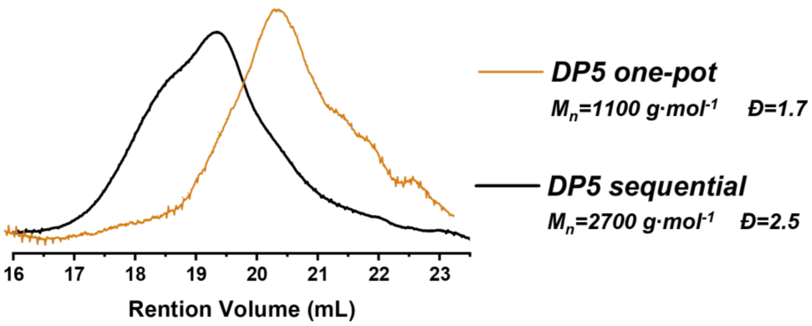

Figure S34. SEC information and traces of DP5 from one-pot synthesis and DP5 from sequential reaction.

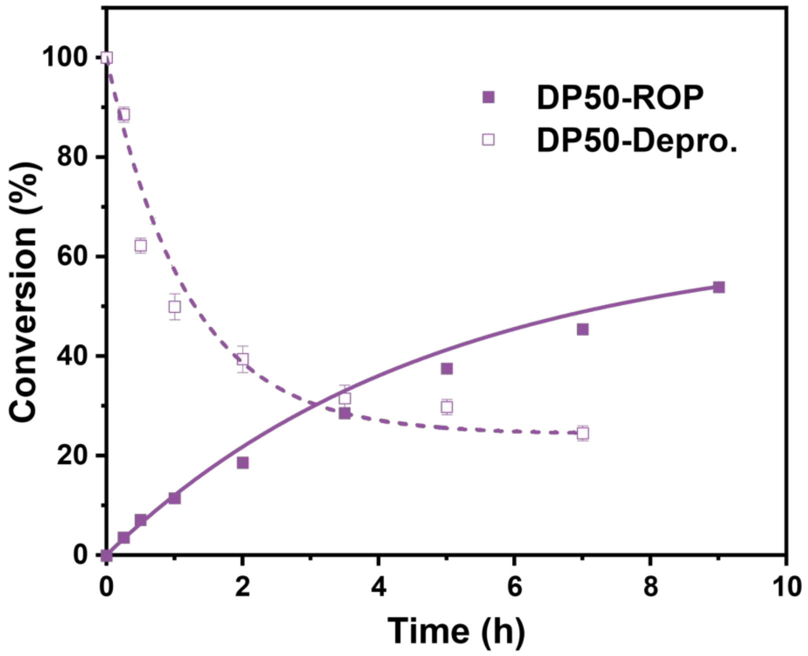

Figure S35. Conversion of the deprotection and ring-open polymerization of DP50.

Table S1. Molecular weight (Da) and degree of polymerization results from $CHCl_3$ SEC, ESI-MS and ^{1}H NMR.

	M_n (SEC)	M_w (SEC)	Đ (SEC)	M_n (ESI-MS)	M_w (ESI-MS)	Đ (ESI-MS)	M_n (NMR)
**DP5**	1100	1900	1.7	520	590	1.1	900
**DP10**	1900	3400	1.9	900	1230	1.4	2000
**DP20**	2800	5700	1.9		/		2500
**DP50**	6500	10200	1.7				3100
**DP5-A**	1200	2800	2.4	730	1080	1.5	1000
**DP10-A**	3400	7200	1.9				1900
**DP20-A**	3000	4300	1.4		/		2500
**DP50-A**	5700	10400	1.8				3200

M_n(ESI-MS), M_W(ESI-MS) and Đ (ESI-MS) were calculated through the formula:

$$M_n = \frac{\sum N_i M_i}{\sum N_i}$$

$$M_w = \frac{\sum N_i M_i^2}{\sum N_i M_i}$$

$$Đ = \frac{M_w}{M_n}$$

The molecular weight and dispersity of DP20 and DP50 were not determined by ESI-MS. For long-chain polymers, ESI-MS measurements are inherently biased toward the low molecular weight region due to the nature of the ionization process.[1] Consequently, for DP20 and DP50, the data obtained from ESI-MS lose their significance.

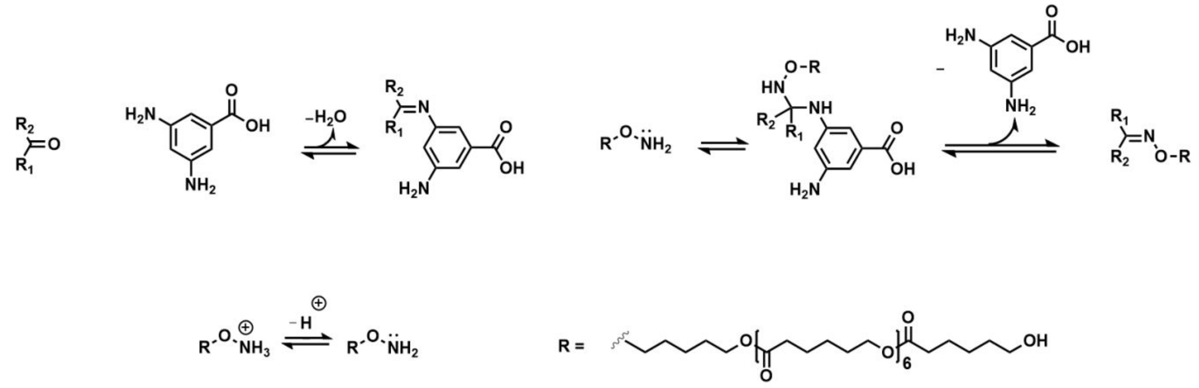

Scheme 1. Mechanistic pathway of oxime ligation under acidic conditions with DABA catalysis.

(1) Nitsche, T.; Sheil, M. M.; Blinco, J. P.; Barner-Kowollik, C.; Blanksby, S. J. Electrospray Ionization-Mass Spectrometry of Synthetic Polymers Functionalized with Carboxylic Acid End-Groups. J Am Soc Mass Spectrom ***2021****, 32 (8), 2123-2134. DOI: 10.1021/jasms.1c00085*
